# Sub-ohm vaping increases the levels of carbonyls, is cytotoxic, and alters gene expression in human bronchial epithelial cells exposed at the air–liquid interface

**DOI:** 10.1186/s12931-020-01571-1

**Published:** 2020-11-19

**Authors:** Alexandra Noël, Ekhtear Hossain, Zakia Perveen, Hasan Zaman, Arthur L. Penn

**Affiliations:** Department of Comparative Biomedical Sciences, School of Veterinary Medicine, Louisiana State University, 1909 Skip Bertman Drive, Baton Rouge, LA 70803 USA

**Keywords:** Electronic nicotine delivery systems (ENDS), Electronic-cigarette, Vaping, Sub-ohm, Carbonyls, Cellular toxicity

## Abstract

**Background:**

Exposure to electronic-cigarette (e-cig) aerosols induces potentially fatal e-cig or vaping-associated lung injury (EVALI). The cellular and molecular mechanisms underlying these effects, however, are unknown. We used an air–liquid interface (ALI) in vitro model to determine the influence of two design characteristics of third-generation tank-style e-cig devices—resistance and voltage—on (1) e-cig aerosol composition and (2) cellular toxicity.

**Methods:**

Human bronchial epithelial cells (H292) were exposed to either butter-flavored or cinnamon-flavored e-cig aerosols at the ALI in a Vitrocell exposure system connected to a third-generation e-cig device. Exposures were conducted following a standard vaping topography profile for 2 h per day, for 1 or 3 consecutive days. 24 h after ALI exposures cellular and molecular outcomes were assessed.

**Results:**

We found that butter-flavored e-cig aerosol produced under ‘sub-ohm’ conditions (< 0.5 Ω) contains high levels of carbonyls (7–15 μg/puff), including formaldehyde, acetaldehyde and acrolein. E-cig aerosol produced under regular vaping conditions (resistance > 1 Ω and voltage > 4.5 V), contains lower carbonyl levels (< 2 μg/puff). We also found that the levels of carbonyls produced in the cinnamon-flavored e-cig aerosols were much lower than that of the butter-flavored aerosols. H292 cells exposed to butter-flavored or cinnamon-flavored e-cig aerosol at the ALI under ‘sub-ohm’ conditions for 1 or 3 days displayed significant cytotoxicity, decreased tight junction integrity, increased reactive oxygen species production, and dysregulated gene expression related to biotransformation, inflammation and oxidative stress (OS). Additionally, the cinnamon-flavored e-cig aerosol induced pro-oxidant effects as evidenced by increases in 8-hydroxy-2-deoxyguanosine protein levels. Moreover, we confirmed the involvement of OS as a toxicity process for cinnamon-flavored e-cig aerosol by pre-treating the cells with N-acetyl cysteine (NAC), an antioxidant that prevented the cells from the OS-mediated damage induced by the e-cig aerosol.

**Conclusion:**

The production of high levels of carbonyls may be flavor specific. Overall, inhaling e-cig aerosols produced under ‘sub-ohm’ conditions is detrimental to lung epithelial cells, potentially via mechanisms associated with OS. This information could help policymakers take the necessary steps to prevent the manufacturing of sub-ohm atomizers for e-cig devices.

## Background

The advent of electronic nicotine delivery systems (ENDS), commonly called electronic-cigarettes (e-cigs), has had a profound effect on cigarette smoking habits in the United States. In 2006, the year before the introduction of ENDS to the United States’ market, 21% of adult Americans were cigarette smokers. By 2018 that percentage had been reduced to 13.7% [1, 2]. While 10 million of the 13 million ENDS users in the United States are adults, 3.5 million ENDS users are preteens and teens [3, 4]. Battery-operated ENDS devices use heat to produce an inhalable aerosol from a liquid mixture of nicotine, flavoring chemicals and humectants [5]. The e-cig aerosol is a complex mixture of fine and ultrafine particles and gases, that contains in addition to nicotine, at least 30 different chemicals, including metals [6, 7]. Although ENDS devices use similar scientific principles to generate an aerosol from an e-liquid, there are significant differences in device configurations between the various generations of ENDS. This creates major challenges for ENDS-related research, as (1) standardized assessments are absent; (2) there are more than 2800 different models of ENDS from 466 identified brands [8]; plus (3) over 7700 unique e-liquid flavors [8]. This is a significant public health concern, since, as demonstrated by the 2019–2020 outbreak in the United States, exposures to ENDS aerosols can induce potentially fatal e-cigarette or vaping-associated lung injury (EVALI) [9]. This clearly demonstrates that little is known regarding the long-term pulmonary effects of inhaling ENDS heated and aerosolized humectants, nicotine as well as flavors.

In dual-users of both conventional cigarettes and e-cigs, use of e-cigs leads to declines in lung function, increased airflow resistance and significantly increased risk of having a myocardial infarction [10, 11]. Inhaling a 2-s e-cig puff can result in airway deposition of 6.25 × 10^10^ particles that can interact with epithelial cells along the entire respiratory tract [12]. Studies of human bronchial epithelial cells exposed to e-cig aerosol at the air–liquid interface (ALI) showed that compared to air-controls, e-cig aerosol decreased cell viability, metabolic activity and ciliary beat frequency, while increasing oxidative stress and the release of inflammatory cytokines, including IL-1β, IL-6, IL-8 and IL-10 [13–15]. Augmented cellular levels of oxidative stress have been linked to the emission of reactive oxygen species (ROS) from e-cig aerosols [16]. While further in vitro studies are necessary to provide insight into the cellular and molecular toxicity induced by e-cig aerosol, the studies to date strongly suggest that ENDS aerosols may not be ‘safe’ for lung cells.

Propylene glycol (PG) and/or vegetable glycerin (VG), the base constituents of e-liquid formulation, are used almost exclusively in all ENDS. While PG and VG are “generally recognized as safe” (GRAS) food additives, their safety for the lungs following aerosolization has not been established. Furthermore, thermal degradation of VG and the chemical interactions of the e-liquid components produce emissions of carbonyls, including formaldehyde and acetaldehyde, known to be potent threats to human health [17, 18]. Unlike first-generation e-cigs, where aerosol levels of toxic chemicals, including formaldehyde (0.02 to 0.37 μg/puff), were up to 600 times lower than those found in cigarette smoke (0.9 to 11.9 μg/puff), second and third-generation e-cig aerosols contain formaldehyde at similar or higher levels (1.8 μg/puff) than those found in cigarette smoke [17, 19–23]. Design features of third-generation e-cigs allow for user adjustment of: (1) atomizer resistance, responsible for heating the e-liquid, and (2) battery voltage, that provides power to the device [8]. The combination of a given resistance and voltage affects e-cig aerosol physicochemical composition. Low resistance combined with high voltage increases the amount of aerosol produced, the intensity of the taste and the ‘throat hit’ [6, 19]. Those user-altered settings are used to create different vaping styles, including sub-ohm vaping or ‘cloud chasing’, popular among younger e-cig users [13]. Sub-ohm atomizers (resistance < 0.5 Ω) produce large exhaled clouds, potentially leading to exposure to elevated levels of carbonyls [24–26]. Besides heating conditions, the composition and constituents’ ratios in the e-liquid also influence chemical levels found in the aerosol, as do effects related to the chemistry of the e-cig flavoring agent. Cinnamaldehyde, the major flavoring chemical found in cinnamon-flavored e-liquids, and diacetyl, associated with butter flavors, are two of the Flavor and Extract Manufacturers Association high-priority flavoring chemicals for respiratory hazard, when inhaled by workers. These flavoring chemicals impair lung function and cause irreversible lung damage (bronchiolitis obliterans, i.e., ‘popcorn lung’) [27]. These user-modifiable factors can significantly impact toxicity of the inhaled e-cig aerosol. Thus, studies (a) examining how e-cig devices’ adjustable components affect aerosol composition and (b) comparing the toxicity of these generated aerosols are urgently required.

The present study was designed to determine the influence of atomizer resistance and battery voltage—on (1) e-cig aerosol composition and (2) cellular toxicity. A physiologically-relevant ALI in vitro model was employed to investigate the effects on lung cells of cinnamon- and butter-flavored e-cig aerosols produced under sub-ohm vaping conditions. Since e-cig use among youth and young adults is rising [4], it is imperative to better understand the characteristics and toxicity of the e-cig aerosols to provide scientific evidence supporting regulations on e-cig device design features and e-liquid manufacturing.

## Methods

### Generation of e-cig aerosols and chemical analysis

The e-liquids composed of 36 mg/mL of nicotine, 50/50 PG/VG ratio, and with either butter or cinnamon flavors were purchased from EC Blend (Medford, OR). The 36 mg/mL concentration of nicotine was selected to mimic the exposures of heavy smokers (> 1 pack of cigarette per day). The e-liquids were analyzed independently for nicotine and propylene glycol content by Bureau Veritas (Buffalo, NY) using gas and liquid chromatography (GC/LC) techniques. The butter-flavored e-liquid had a nicotine content of 36.0 mg/mL and the PG concentration was 44.1%, while the cinnamon-flavored e-liquid had a nicotine content of 37.1 mg/mL and the PG concentrations was 31.8%. The e-liquids were aerosolized by a Scireq® (Montreal, QC, Canada) third-generation e-cig generator, as we previously described [28]. The e-cig device was operated with three different atomizers (resistance of 0.15, 0.5 and 1.5 Ω) and different battery voltages (2.8, 3.8 and 4.8 V), which represent e-cig devices’ operational settings that are accessible to e-cig users. This yields a total of 9 distinct heating conditions that were evaluated for e-cig aerosol composition [29]. Vaping was conducted under a topography profile of 3-s puff duration, and a 55-mL puff volume every 30-s. This vaping regime is in accordance with the method recommended by the Cooperation Centre for Scientific Research Relative to Tobacco (CORESTA) CRM N° 81 [30]. E-cig aerosol chemical composition characterization was performed as per the method described in Flora et al. [31]. Briefly, 10-puff e-cig aerosol samples were collected in our laboratory. Samples for nicotine, glycerin and propylene glycol were collected on 44-mm Cambridge filter pads at a loading regimen of 1 L/min. Quantification (Enthalpy Analytical, LLC, Durham, NC) was by gas chromatography with a flame ionization detector (GC-FID). Samples for carbonyls were collected in 2,4-dinitrophenylhydrazine (DNPH) tubes at a loading regimen of 1 L/min and were subsequently analyzed (Enthalpy Analytical, LLC) using the EPA method TO-11A based on high performance liquid chromatography (HPLC). All samples were collected on site at the Inhalation Research Facility at Louisiana State University and were shipped overnight on dry ice to Enthalpy Analytical, LLC.

### Cell culture

We exposed human bronchial-epithelial cell line (NCI-H292, H292; American Type Culture Collection, ATCC CRL-1848, Manassas, VA) to e-cig aerosols. We selected H292 cells based on their life span, passage and stability, for which they are frequently used as a model system for ALI exposures, particularly for studying long-term toxicity of aerosols [14, 32, 33]. H292 cells were grown in RPMI 1640 medium containing 10% FBS, penicillin (100 U/ml) and streptomycin (100 mg/ml) at 37 °C in a humidified 5% CO_2_ incubator. H292 cells (1–2 × 10^5^/mL) were seeded on 24 mm transwells with a 0.4-μm pore size polyester membrane insert (Corning) and inserted into 6-well culture plates. Cells were maintained on RPMI-1640-supplemented medium (ATCC 30-2001). Before initiation of differentiation at the ALI, we supplied both the apical and basal chambers with serum-free medium [32]. Differentiation was initiated by removing the apical medium [34, 35]. For H&E staining of the cells, the transwell inserts were first embed in agarose as per STEMCELL Technologies Product procedures, and then paraffin-embedded and sectioned (5 μm thin). H&E staining was performed using standard protocols [36]. All experiments were performed with cells from passages 2 to 6. This cell line grown in our laboratory was authenticated by ATCC using the short tandem repeat (STR) technique, following ISO 9001:2008 and ISO/IEC 17025:2005 quality standards. The cells showed an exact match for the CRL-1848 cells of the ATCC STR database.

In addition, to evaluate the implication of oxidative stress-induced e-cig aerosol responses, sub-groups of H292 cells grown at the ALI were pre-treated with N-Acetyl-L-cysteine (NAC) (Sigma-Aldrich) at a concentration of 5 mM, 12 h before the ALI exposure to either medical grade compressed air or e-cig aerosols.

### Air–liquid interface (ALI) exposures

The ALI exposures allow cells to be exposed to all the aerosol components, including the particulate and gas phases. We used a customized ALI exposure system from Vitrocell Systems GMBH (Waldkirch, Germany) that enables direct exposure of cells to various aerosols. Our customized ALI system is composed of a Vitrocell 6/4 stainless steel exposure module for 4 × 6 well/24 mm diameter inserts, which is connected to a distribution system for the Vitrocell 6 modules. This exposure system is also equipped with a quartz crystal microbalance (QCM) sensor for Vitrocell 6, which has a performance resolution 10 ng/cm^2^ per second. This is in addition to a Vitrocell 6/3 stainless steel exposure module for 3 × 6 well/24 mm diameter inserts, which is connected to a clean air distribution system for air control-exposed cells. We used medical grade compressed air to supply our clean air distribution system, and thus this air was used for aerosol dilution and for our control group exposures. Overall, the exposure modules are composed of seven chambers: four for e-cig aerosol exposures, including one chamber with the QCM, and three for medical grade compressed air exposures.

To study the effect of butter- and cinnamon-flavored e-cig aerosols on cellular toxicity of H292 cells, we connected the third-generation e-cig device (Scireq®), operating with the device settings and topography profile described above, to the Vitrocell ALI exposure system. The cells were seeded on distinct transwell inserts, which were independently grown for 21 days at the ALI. During each experiment, 3 cell inserts were randomly assigned to a different treatment, either e-cig aerosols (n = 3) or medical grade compressed-air (n = 3), and then exposed simultaneously to the respective test atmosphere via our in vitro inhalation exposure system. For scientific rigor, the same experiment was performed independently on three separate occasions (which were done on 3 different days). Also, we used 2 different medical grade compressed air control groups, one for each flavored e-cig aerosols. Diluted with 1 L/min of medical grade compressed air, the e-cig aerosol concentrations were measured with the QCM placed inside the cell chamber. While the cells were directly exposed in the ALI exposure system, warm water (36–37 °C) was circulated around the chambers via a water bath. The exposure chambers were cleaned after each exposure.

H292 cells were exposed to either e-cig aerosols or medical grade compressed air for 2 h per day for 1 or 3 consecutive days. After the last exposure, cells were incubated at 37 °C for 24 h and biological endpoints were measured.

### Scanning electron microscopy (SEM)

Representative air-control, butter- and cinnamon-flavored e-cig aerosol-exposed cells were processed by SEM techniques. In brief, cells on the transwell inserts were fixed (1.25% (v/v) glutaraldehyde + 2% formaldehyde in 0.1 M sodium cacodylate buffer, pH 7.4) immediately upon completion. Then, the membranes were detached from the insert and dehydrated with ethanol. Additional dehydradation was applied to each sample by incubation with hexamethyldisilizane, and then samples were placed overnight in a dessicator. The membranes were cut from the inserts and mounted on standard specimen mounts. Samples were examined with an FEI Quanta 3D scanning electron microscope (SEM) at an accelerating voltage of 5 kV.

### Trypan-blue dye exclusion assay

24 h after the last exposure, cell viability was measured by the trypan-blue dye-exclusion assay. An aliquot of 10 μl of cell suspension was pipetted into a TC10 counting slide (catalog #1450015, Bio-Rad Laboratories, Hercules, CA) and placed in the TC20 automated cell counter (catalog #1450102, Bio-Rad Laboratories, Hercules, CA). The cell counter provides total and viable cells counts. All samples were run in duplicate.

### Extracellular lactate dehydrogenase (LDH) measurements

Cytotoxicity was determined by measuring the levels of LDH in the cell culture medium using a commercially available assay kit (CyQUANT™ LDH Cytotoxicity Assay Kit, Catalog # C20300, Invitrogen, Thermo Fisher Scientific, Waltham, MA), as per the manufacturer’s instructions. The assay was conducted in duplicate for each sample. 50 μL of cell culture medium, which was removed from the basal side of the Transwell, was combined with the LDH assay reactive mixture in a 96-well plate. Subsequently, the absorbance was read at 490/680 using a spectrophotometric plate reader (Tecan Infinite 2000, Tecan Group Ltd, Mannedorf, Switzerland). For each sample, the cell medium absorbance was normalized to the total cell count. Absorbance values for the air control groups were set at 100%. Samples were run in triplicate.

### Extracellular ROS measurements

Extracellular ROS production was detected using the OxyBURST Green assay (dihydro-2′,4,5,6,7,7′-hexafluorofluorescein-BSA (H_2_HFF), Catalog # D2935, Invitrogen, Thermo Fisher Scientific, Waltham, MA). H_2_HFF-BSA was dissolved in PBS to obtain a stock concentration of 1 mg/mL, which was protected from light and immediately used. 50 µL of cell culture medium, which was removed from the basal side of the Transwell, was incubated with an equal volume of the H_2_HFF-BSA reagent at room temperature for 15 min. We followed the manufacturer’s instructions. Fluorescence was read using a spectrophotometric plate reader (Tecan Infinite 2000, Tecan Group Ltd, Mannedorf, Switzerland) at an excitation and emission wave length of 497/527, respectively. For each sample, the cell medium fluorescence was normalized to the total cell count. Fluorescence values for the air control groups were set at 100%. Samples were run in triplicate.

### Detection of DNA damages

DNA damage was quantified with the 8-hydroxy-2-deoxyguanosine (8-OHdG) ELISA Kit (catalog # ab201734, Abcam, Cambridge, MA), according to manufacturer’s recommendations. 8-OHdG was measured in cell culture medium, which was removed from the basal side of the Transwell. The ELISA plate was read using a Tecan Infinite 2000 plate reader (Tecan Group Ltd, Mannedorf, Switzerland). 8-OHdG is used as a biomarker of DNA oxidation. For each sample, the 8-OHdG protein concentration was normalized to the total cell count. Samples were run in triplicate.

### Extracellular NO measurements

The NO concentrations in the H292 cell culture media were determined by the Griess reagent assay (catalog #30,100, Biotum, Fremont, CA). 50 µL of the cell culture medium was removed from the basal side of the Transwell and incubated with an equal volume of the Griess reagent at room temperature for 15 min. Sodium nitrite was used to generate a standard curve. We followed the manufacturers’ instructions. The absorbance was determined with a Tecan Infinite 2000 spectrometer (Tecan Group Ltd, Mannedorf, Switzerland) at a wavelength of 490 nm. For each sample, the cell medium absorbance was normalized to the total cell count. Absorbance values for the air control groups were set at 100%. Samples were run in triplicate.

### Transepithelial electrical resistance (TEER) measurement

The integrity of tight junctions between cells was assessed immediately after exposure by TEER using a Millicell-ERS voltohmmeter (Millipore, Burlington, MA). Cell culture medium was added to the surface of the H292 cells grown at the ALI. A background reading of the cell culture medium alone was taken and subtracted from each raw resistance value. The corrected values were multiplied by the surface area of the cell insert and used to calculate the mean Ω*cm^2^. Measurements for each cell insert were made in triplicate. For each sample, the average TEER value was normalized to the total cell count.

### RNA extraction, cDNA synthesis, and quantitative RT-PCR

Isolation and processing of RNA from pooled H292 cells were performed according to procedures described previously [37]. Briefly, total RNA was isolated by a TRIzol/chloroform extraction, followed by column purification with the Qiagen RNeasy Mini Kit. RNA concentrations were measured with a ND-1000 Spectrophotometer (NanoDrop, Wilmington, DE). Isolated pure RNA was reverse transcribed using iScript cDNA Synthesis kit (Bio-Rad Laboratories, Hercules, CA). The resulting cDNA was amplified in a T100 Thermal Cycler (Bio-Rad Laboratories, Hercules, CA). Quantitative real-time PCR was performed on cDNA samples from H292 cells with either inventoried TaqMan gene expression assays primers-probe sets (Applied Biosystems) or designed primers listed and described in Additional file 1: Table S1. Reaction volumes were 25 µL and reaction cycles were performed for each gene in an Applied Biosystems 7300 Real Time PCR System. Fold changes were calculated using the 2−ΔΔCt method. *β-ACTIN* was the housekeeping gene used for normalization. Results are reported as fold-change over air-control group. A fold-change > ± 1.5 was considered significant.

### Statistical analysis

All statistical analyses were performed using Microsoft Excel or GraphPad Prism 7 software. Data are expressed as means ± standard error of the mean (SEM). Data are also expressed as percent change relative to the respective air control group, set at 100%. Statistically significant differences between groups were analyzed using either a Student-*t* test or a one-way analysis of variance (ANOVA) followed by a Tukey's post-hoc test, when testing 3 or more groups. Statistical significance was achieved with a p-value < 0.05.

## Results

### Sub-ohm vaping increases the levels of carbonyls in butter-flavored e-cig aerosols

**Fig. 1:**
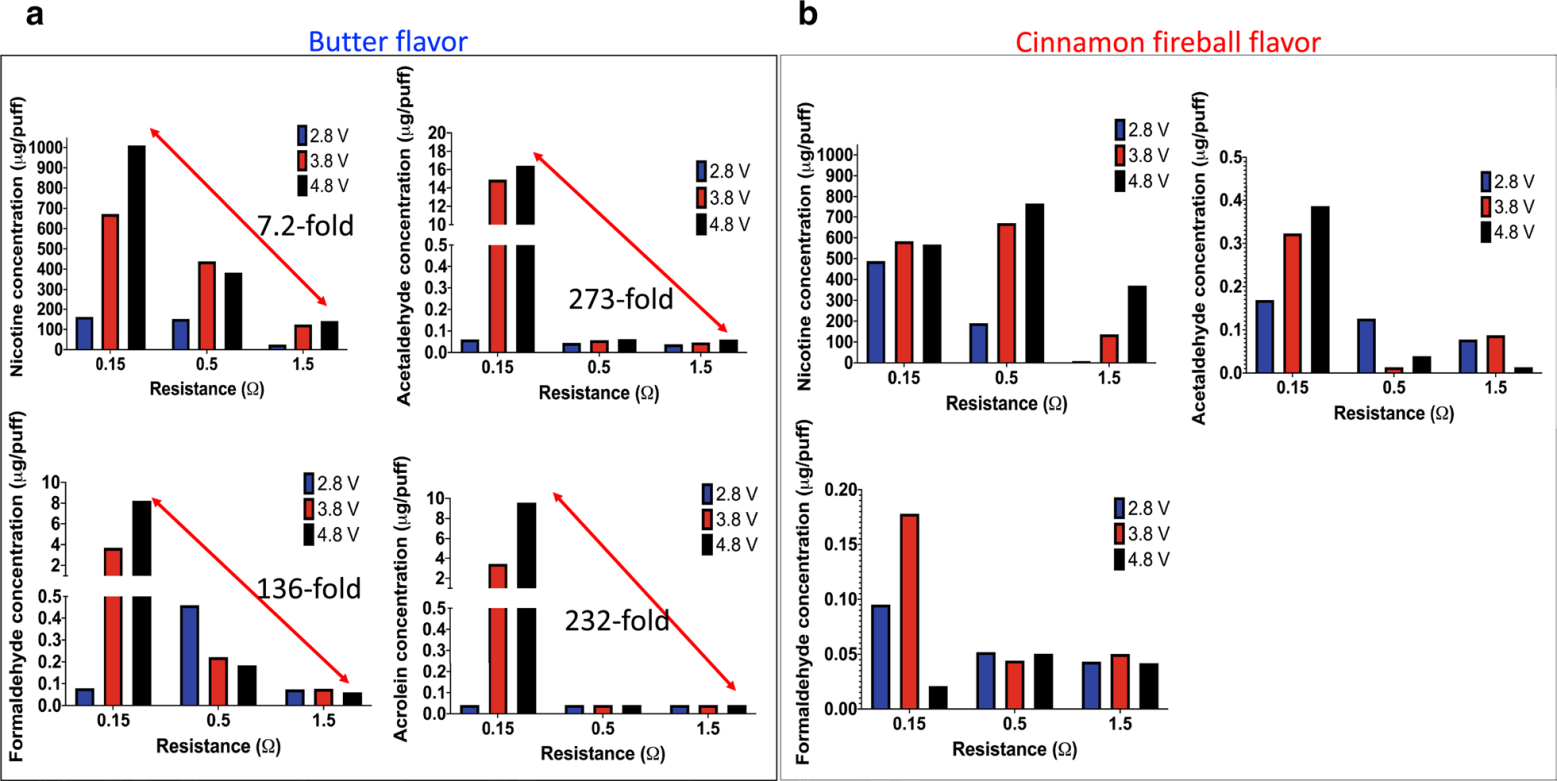
Sub-ohm vaping increases the levels of carbonyls in butter-flavored e-cig aerosols. The e-cig device was operated with three different atomizers (resistance of 0.15, 0.5 and 1.5 Ω) and different battery voltages (2.8, 3.8 and 4.8 V), which yield a total of 9 distinct heating conditions that were evaluated for e-cig aerosol nicotine and carbonyls content. Vaping was conducted under a topography profile of 3-s puff duration, and a 55-mL puff volume every 30-s. 10 puffs of each e-cig aerosol were collected on site at the Inhalation Research Facility at Louisiana State University and were shipped overnight on dry ice to Enthalpy Analytical, LLC for subsequent chemical analysis. **a** Concentrations (µg/puff) of nicotine, acetaldehyde, formaldehyde and acrolein in butter-flavored e-cig aerosols. **b** Concentrations (µg/puff) of nicotine, acetaldehyde and formaldehyde in cinnamon-flavored e-cig aerosols. Acrolein was below the limit of detection in the cinnamon-flavored e-cig aerosols. This initial profile screening was comprised of a one-time chemical analysis of 18 different e-cig aerosol samples

We found that high voltage sub-ohm vaping significantly increases the levels of acetaldehyde, acrolein and formaldehyde present in butter-flavored e-cig aerosols. The results in Fig. 1a for butter-flavored e-cig aerosols demonstrate that for a given voltage, i.e. 2.8, 3.8 or 4.8 V, the lower the atomizer’s resistance (0.15 vs. 1.5 Ω) the higher the levels of nicotine and carbonyls in the aerosols. For these aerosols, nicotine, acetaldehyde, formaldehyde, and acrolein levels were 7.2-, 273-, 136-, and 232-fold higher, respectively, when an atomizer of 0.15 vs. 1.5 Ω was used with a battery voltage set at 4.8 V (Fig. 1a). In contrast, we found that for a given resistance greater than 0.5 Ω, increasing the voltage applied to the e-cig device did not particularly affect the concentration of carbonyls produced (Fig. 1a). Increasing the voltage used with sub-ohm (0.15 Ω) resistance, however, increases the concentration of nicotine and carbonyls in a voltage-dependent manner (Fig. 1a). Results for cinnamon-flavored e-cig aerosols showed concentrations of nicotine and carbonyls that were lower than those obtained for the butter-flavored aerosols (Fig. 1b). For instance, the concentration of acrolein in the cinnamon-flavored e-cig aerosols were below the limit of detection. Overall, these data suggest that high production of carbonyls under sub-ohm conditions may be flavor-specific.

### The presence of cilia at the surface of H292 cells confirms differentiation at the ALI

**Fig. 2:**
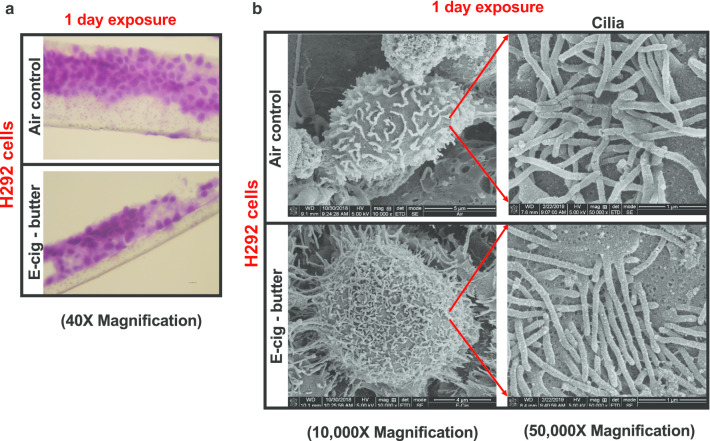
The presence of cilia at the surface of the cells confirms that H292 cells were grown at the air–liquid interface (ALI). **a** Characterization of the ALI cell culture model. H&E staining reveals the multiple cell layers on the apical surface following 1 day of exposure to either air or butter-flavored e-cig aerosol. **b** Scanning electron microscopy images of representative H292 cells exposed to either air or butter-flavored e-cig aerosols for 1 day at the ALI, with a higher magnification of the cilia present at the surface of the cells. Images showed are representative for each exposure group. For air-exposed cells: a total of 38 SEM images were taken; for butter-flavored e-cig aerosol-exposed cells: a total of 53 SEM images were taken

H292 cells were grown on a transwell insert and differentiated for 21 days at the ALI. Cells were confluent and this resulted in a pseudostratified bronchial epithelium (Fig. 2a). The presence of cilia at the surface of the cells confirms differentiation at the ALI (Fig. 2b). Cell morphology changes were observed qualitatively by SEM following 1-day of exposure to butter-flavored e-cig aerosol, with cells appearing to display disorganized ciliated arrangements at their surface (Fig. 2b).

### 3 Days of butter-flavored e-cig aerosol exposure under sub-ohm conditions decreases viable cell numbers and dysregulates gene expression to a greater extent than under regular vaping conditions

**Fig. 3.**
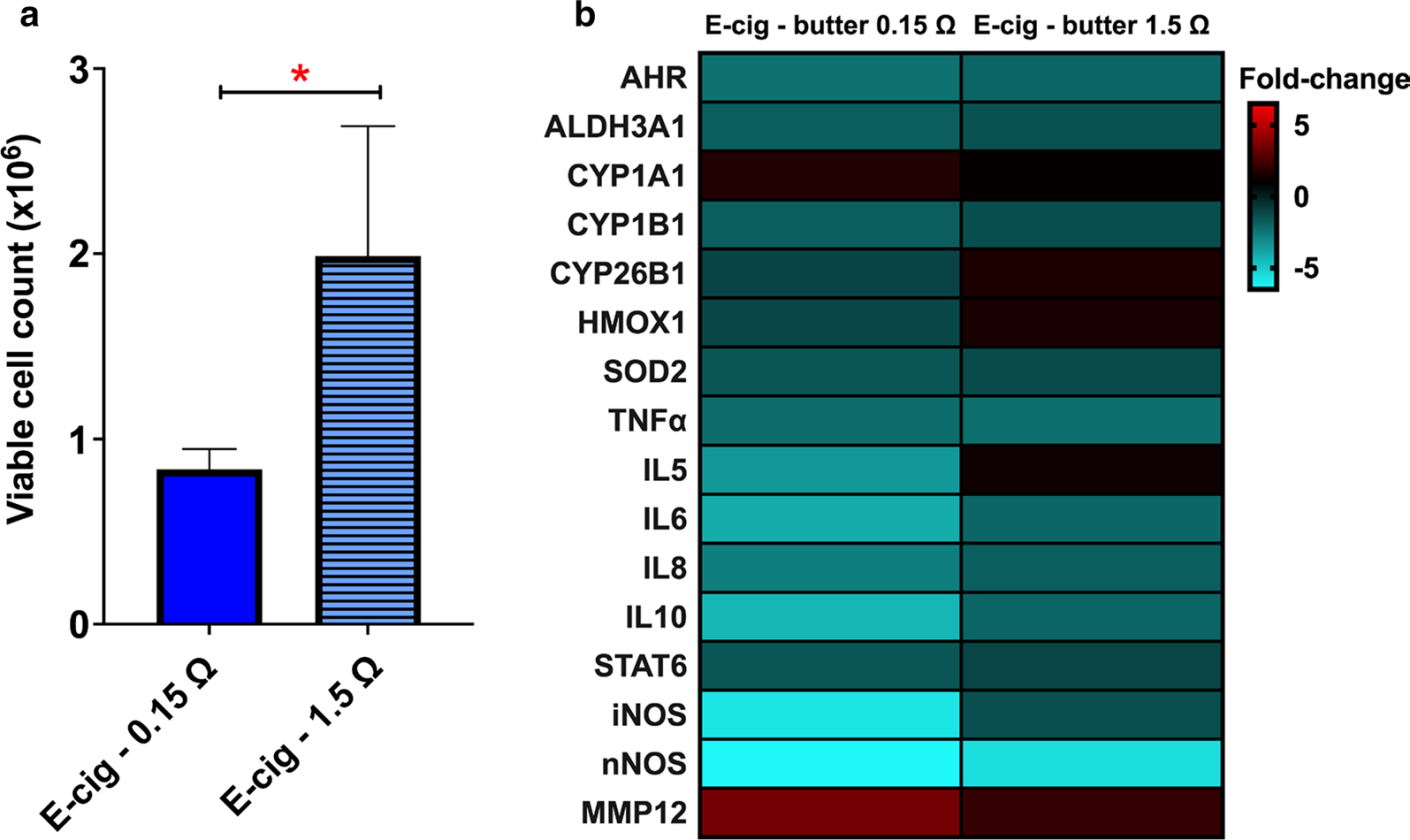
3 Days of butter-flavored e-cig aerosol exposure under sub-ohm conditions decrease viable cell numbers and dysregulate gene expression to a greater extent than under regular vaping conditions. Butter-flavored e-cig aerosols were produced either under sub-ohm or regular vaping conditions and H292 cells were exposed to these aerosols at the air–liquid interface (ALI) for 3 days. **a** Numbers of viable cells were significantly decreased by the e-cig aerosol produced under sub-ohm vaping conditions. Data are presented as mean ± SEM. Comparisons between groups were made by the student t-test; *p < 0.05: significantly different from the other group. Data are from one experiment representative of results from three independent experiments, each performed in triplicate (n = 3 per group). For each cell insert, bioassays were further evaluated in duplicates. **b** Heatmap of dysregulated genes reveals that more genes are dysregulated by the e-cig aerosol produced under sub-ohm conditions compared to regular vaping conditions. Data are from H292 cells from distinct experiments. Data are presented as fold-change over the respective air-control group. Fold-changes > ± 1.5 were considered significant. Results from other independent experiments are represented in Additional file 1: Figure S1.

Since butter-flavored e-cig aerosols generated under sub-ohm (0.15 Ω) conditions produced higher levels of toxic chemicals than regular vaping conditions (1.5 Ω) (Fig. 1a), next we investigated whether this difference in vaping conditions would translate into toxicological responses of lung cells. The number of viable H292 cells decreased by more than 50% following exposure to the aerosol product of sub-ohm vaping (Fig. 3a). Figure 3b shows that exposure to butter-flavored e-cig aerosol generated with an atomizer of 1.5Ω, dysregulated the expression of 9 genes: 6 genes (*AHR*, *TNF-α*, *Il-6*, *IL-8*, *IL-10*, and *nNOS*) were down-regulated and 3 genes (*CYP26B1*, *HMOX1*, *MMP12*) were up-regulated. In contrast, exposure to sub-ohm-generated e-cig aerosol resulted in down-regulation of 14 genes, 6 of which (*AHR*, *TNF-α*, *IL-6*, *IL-8*, *IL-10*, and *nNOS*) were also down-regulated at 1.5 Ω, in addition to five more genes (*ALDH3A1*, *CYP1B1*, *SOD2*, *IL-5*, *iNOS* and *STAT6*). Only two genes were up-regulated by sub-ohm vaping (*CYP1A1* and *MMP12*). The down-regulation of genes associated with inflammatory cytokines by e-cig aerosols that was observed 24 h post the last exposure may be due to the time-course response profile of cytokines following acute injury [38]. At 24 h post-exposure, we may have missed the peak of an acute phase response, which can occur 1 to 4 h after injury [38]. The 7 genes that were affected by both exposure conditions (0.15 Ω & 1.5 Ω) were dysregulated to a greater extent following exposure to e-cig aerosols produced under sub-ohm conditions (Fig. 3B). This suggests that sub-ohm vaping may be more detrimental to lung health than vaping under regular conditions.

### 1 Day of e-cig aerosol exposure under sub-ohm conditions is cytotoxic, alters gene expression and levels of reactive oxygen species, with cinnamon-flavored e-cig aerosol being more potent

**Fig. 4.**
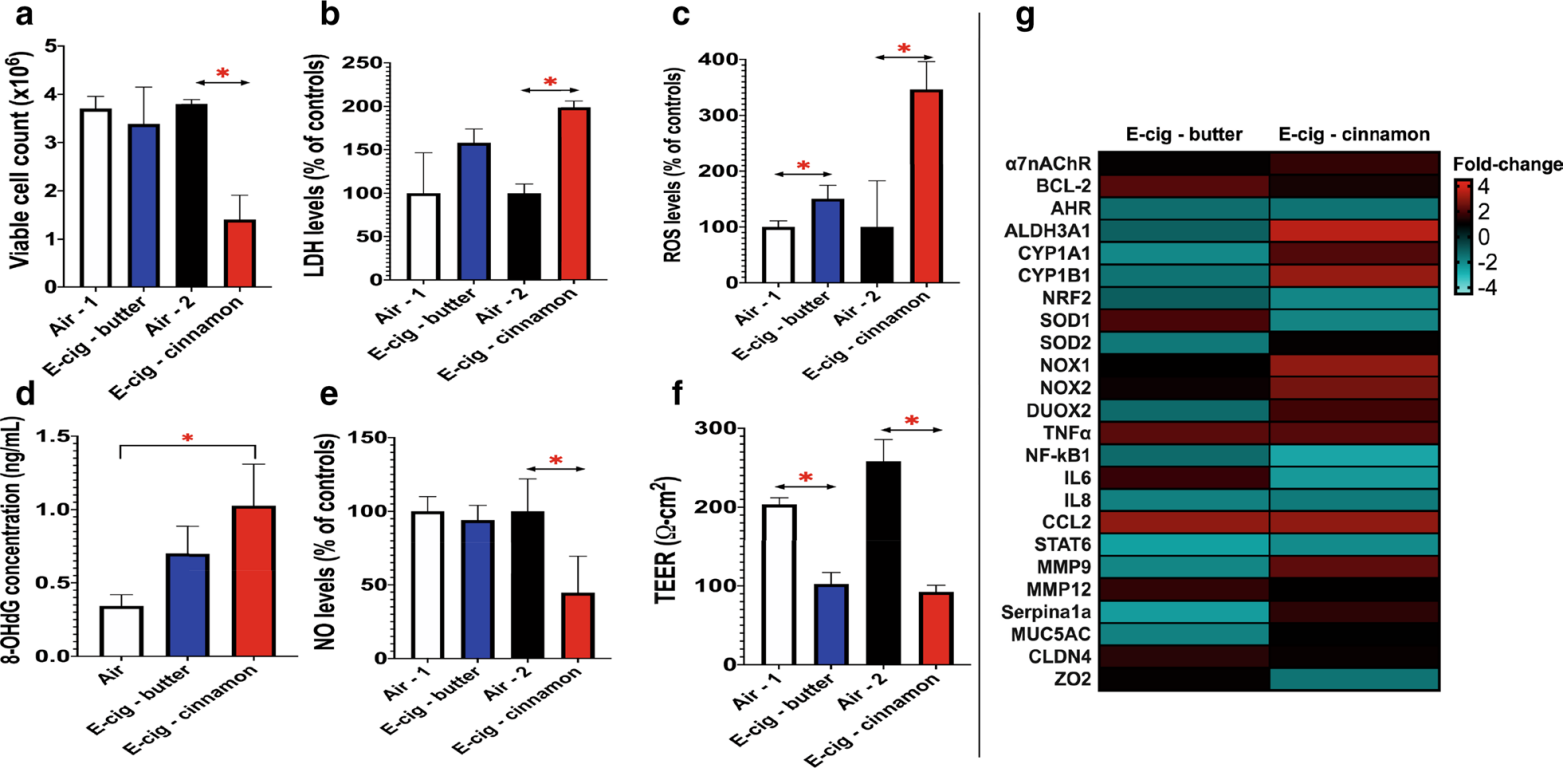
1 Day of e-cig aerosol exposure under sub-ohm conditions (0.15 Ω & 4.8 V) affects the integrity of H292 cells’ tight junctions and revealed that cinnamon flavor is more cytotoxic and causes oxidative damage to a greater extent than butter flavored-e-cig aerosol. H292 cells were exposed to either butter- or cinnamon-flavored e-cig aerosol at the air–liquid interface (ALI) for 1 day. **a** Numbers of viable cells were significantly decreased by the cinnamon-flavored e-cig aerosol compared to respective air control. **b** Levels of extracellular lactate dehydrogenase (LDH) were significantly increased by the cinnamon-flavored e-cig aerosol compared to respective air control. **c** Levels of extracellular reactive oxygen species (ROS) were significantly increased by both butter- and cinnamon-flavored e-cig aerosols compared to their respective air control groups. **d** The extracellular protein concentration of 8-OHdG was significantly increased by the cinnamon-flavored e-cig aerosol compared to the air control group. This ELISA was analyzed for cell inserts (n = 3 per group). Each cell insert was further evaluated in duplicate. **e** Levels of extracellular nitric oxide (NO) were significantly decreased by the cinnamon-flavored e-cig aerosol compared to respective air control. **f** The transepithelial electrical resistance (TEER) values were significantly decreased by both butter- and cinnamon-flavored e-cig aerosols compared to their respective air control groups. For assays (**a, b, c, f**), data are from one experiment representative of results from three independent experiments, each performed in triplicate (n = 3 per group). For each cell insert, bioassays were further evaluated in duplicate or triplicate. For all assays (**b–f**), data were normalized to cell count and are presented as mean ± SEM. Comparisons between e-cig group and respective air control group were made by the student t-test; *p < 0.05: significantly different from respective air control. **g** Heatmap of the dysregulated genes by the e-cig aerosol exposures. Data are from H292 cells from distinct experiments. Data are presented as fold-change over the respective air-control group. Fold-changes > ± 1.5 were considered significant. Results from other independent experiments are represented in Additional file 1: Figures S2 to S5

Since exposures to butter-flavored e-cig aerosols under sub-ohm (0.15 Ω) conditions were more toxic to lung epithelial cells than exposures under regular vaping conditions (Fig. 3), we next examined whether flavorings could also affect cellular responses, by comparing the in vitro toxicity of butter- and cinnamon-flavored e-cig aerosols produced under sub-ohm conditions. The cell-deposited doses were similar between both treatments, 48.04 μg/cm^2^ ± 20.8 and 47.96 μg/cm^2^ ± 17.7, for the butter-flavored and cinnamon-flavored e-cig aerosols, respectively. While the nicotine concentration in the butter-flavored e-cig aerosol was approximately twice as high as that of the cinnamon-flavored e-cig aerosol (Fig. 1), gene expression of the alpha 7 nicotinic acetylcholine receptor, α7nACh, measured 24 h after the e-cig aerosol exposure, which was used as a biomarker of nicotine exposure, was increased by 1.7-fold only in the cinnamon-flavored e-cig aerosol exposed cells (Fig. 4g). Since all acetylcholine receptors (AChR) can be desensitized after persistent exposures to high levels of agonists [39], it is possible that the high nicotine concentration from the butter-flavored e-cig aerosol desensitized the α7nAChR of the bronchial epithelial cells exposed to this aerosol, which translated into baseline expression of the α7nAChR gene. In addition, we found that although butter-flavored e-cig aerosols contained higher levels of carbonyls than their cinnamon counterparts (Fig. 1), when compared to their respective air control group, 1-day exposure to cinnamon-flavored e-cig aerosols is more cytotoxic than a 1-day exposure to butter-flavored e-cig aerosols, evidenced by a significant decrease in viable cell number (Fig. 4a). Increased cell death includes both necrotic and apoptotic cells. The increased cytotoxicity for the cinnamon-flavored e-cig aerosol exposed-cells (Fig. 4a) is supported by significantly elevated levels of LDH found in their cell culture medium (Fig. 4b). Since LDH release into the cell culture medium is an indicator of increased cell membrane damge, LDH is a marker of cytotoxicity for cells that are thought to be mainly necrotic [40]. BCL-2, a protein that has anti-apoptotic properties, prevents the increased permeability of membranes [41, 42]. We found that the gene expression of *BCL-2* was up-regulated by 2.2-fold following exposure to the butter-flavored aerosol, whereas no change was observed for this gene after exposure to the cinnamon-flavored aerosol (Fig. 4g). Increased expression of *BCL-2* has been reported to prevent apoptotic cell death [41]. This suggests that there may be differences in the mechanism of cell death between these 2 flavored e-cig aerosols. The levels of ROS in the cell culture medium of both exposure groups were increased compared to respective air controls (Fig. 4c). *NRF2* gene expression, a transcription factor activated by ROS that is central to the antioxidant response element (ARE) of cells [43], was down-regulated 1.9-fold in the cinnamon-flavored exposure group (Fig. 4g). *NRF2* downstream genes include the super oxide dismutases (SODs) antioxidant enzymes [43]. *SOD1* were also down-regulated 1.9-fold in the cinnamon exposure group (Fig. 4g). In addition, NADPH-related oxidases, NOX/DUOX, have been implicated in host defense and in the regulated production of ROS for redox-dependent signaling pathways [44]. The gene expression of *NOX1*, *NOX2* and *DUOX2* were up-regulated by 3.0-, 2.6-, and 1.9-fold, respectively, in the cinnamon-flavored e-cig aerosol exposure group (Fig. 4g). Furthermore, 8-OHdG concentration, a biomarker of oxidative DNA damage, was significantly elevated only in the cinnamon-flavored e-cig aerosol exposed-cells (Fig. 4d). This suggests that cinnamon-flavored e-cig aerosol may induce oxidative-stress-mediated lung cell injury to a greater extent than butter-flavored e-cig aerosol. In accordance with the elevated levels of ROS found in the cell culture medium of cells exposed to the cinnamon-flavored e-cig aerosol, we observed a significant reduction in the levels of NO for this exposure group (Fig. 4e). Moreover, the integrity of cellular tight junctions measured by TEER was significantly lower in both exposure groups compared to respective air controls (Fig. 4f). This was further supported by the dysregulation of Claudin4 (*CLDN4*) and the tight junction protein 2 (*ZO2*) in exposed cells (Fig. 4g). The butter-flavored e-cig aerosol up-regulated the expression of *CLDN4* by 1.5-fold, while cinnamon-flavored e-cig aerosol down-regulated *ZO2* expression by 1.5-fold (Fig. 4g). These 2 genes code for proteins that are integral to membranes of epithelium tight junction [45]. Dysregulation of these proteins, including up- and down-regulation, modifies paracellular permeability [45]. This suggest that compromised epithelial cells’ tight junction integrity may be linked with increased ROS for butter- and cinnamon-flavored e-cig aerosols (Fig. 4c), in addition to increased cytotoxicity for the cinnamon exposure group (Fig. 4a, b). With the up-regulation of 11 genes (Fig. 4g), the data suggest that cinnamon-flavored aerosols may be pro-oxidant. Aside from the carbonyls produced in the e-cig aerosol (Fig. 1), this may be due to other factors, including cinnamaldehyde, an e-cig flavoring chemical showed to be toxic in vitro and in vivo [36, 46–50].

### 1 Day of e-cig aerosol exposure-induced oxidative stress-mediated lung cell injury is alleviated by n-acetyl cysteine (NAC)

**Fig. 5.**
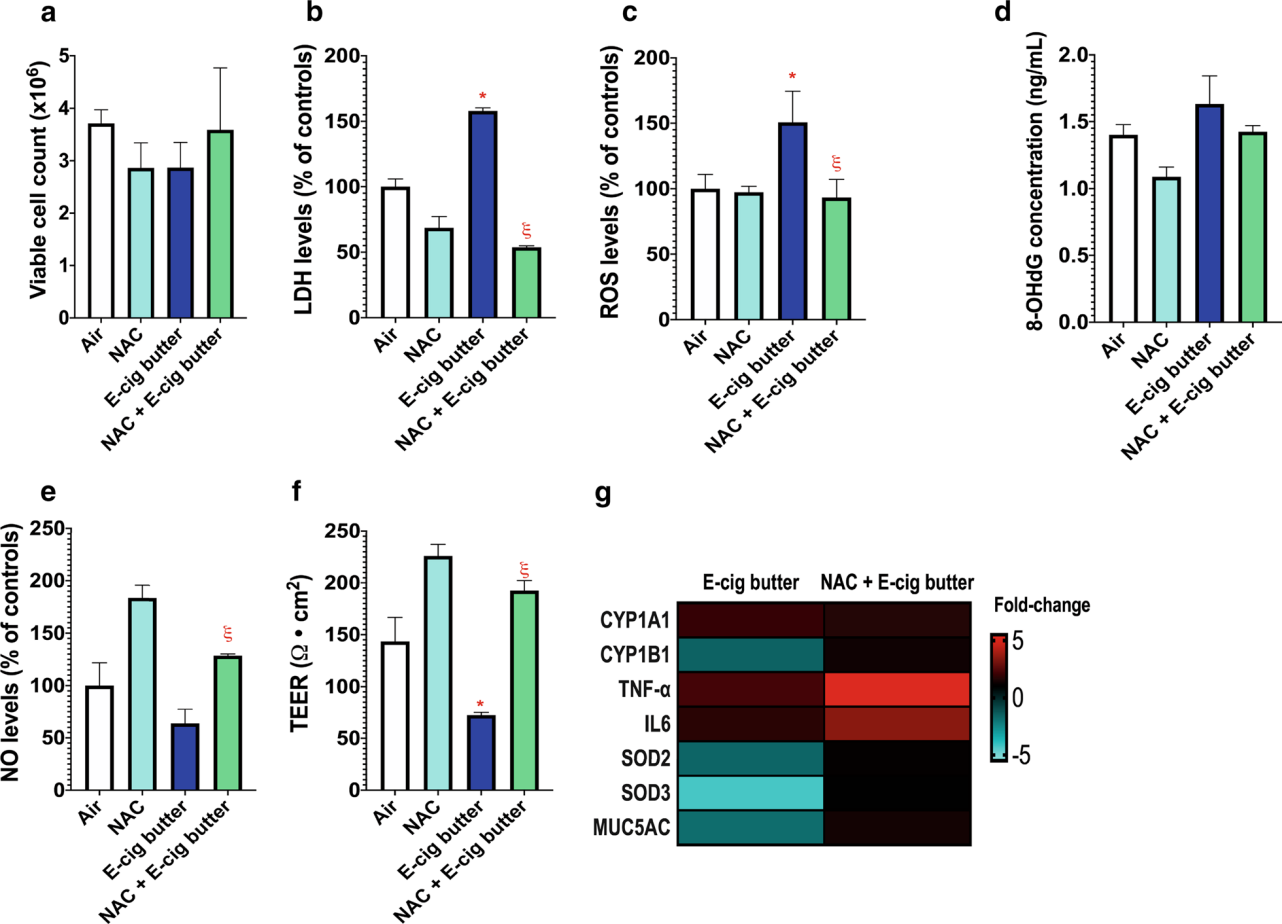
1 Day of butter-flavored e-cig aerosol exposure under sub-ohm conditions-induced oxidative stress-mediated lung cell injury is alleviated by n-acetyl cysteine (NAC) pre-treatment. H292 cells were pre-treated with or without NAC at a concentration of 5 mM for 12 h before exposure to butter-flavored e-cig aerosol for 1 day at the air–liquid interface (ALI). **a** Exposure to butter-flavored e-cig aerosol and NAC pre-treatment had no significant effect on cellular viability. **b** Levels of extracellular lactate dehydrogenase (LDH) were significantly increased by the butter-flavored e-cig aerosol compared to its respective air control; NAC pre-treatment prevented this elevation. **c** Levels of extracellular reactive oxygen species (ROS) were significantly increased by the butter-flavored e-cig aerosol compared to its respective air control; NAC pre-treatment allowed ROS to remain at control levels. **d** Exposure to butter-flavored e-cig aerosol and NAC pre-treatment had no significant effect on the extracellular protein concentration of 8-OHdG. This ELISA was analyzed for cell inserts (n = 3 per group). Each cell insert was further evaluated in duplicate. **e** Levels of extracellular nitric oxide (NO) were not significantly changed by the butter-flavored e-cig aerosol compared to its respective air control; however, the NAC pre-treatment significantly elevated the levels of NO compared to the e-cig exposed-cells that were not pre-treated with NAC. **f** The transepithelial electrical resistance (TEER) values were significantly decreased by the butter-flavored e-cig aerosol compared to its respective air control, and NAC pre-treatment significantly improved the TEER values. For assays (**a**, **f**), data are from one experiment representative of results from three independent experiments, each performed in triplicate (n = 3 per group). For each cell insert, bioassays were further evaluated in duplicate or triplicate. For all assays (**b–f**) data were normalized to cell count and are presented as mean ± SEM. Comparisons between groups were made with one way ANOVA followed by the Tukey's Multiple Comparison Test. *p < 0.05: significantly different from respective air control; ^ξ^*p* < 0.05: significantly different from e-cig aerosol exposed-cells without NAC pre-treatment. **g** Heatmap of dysregulated genes in H292 cells pre-treated with or without NAC followed by exposure to butter-flavored e-cig aerosol. Data are from H292 cells from distinct experiments. Data are presented as fold-change over the respective air-control group. Fold-changes > ± 1.5 were considered significant. Results from other independent experiments are represented in Additional file 1: Figures S2 and S3

**Fig. 6.**
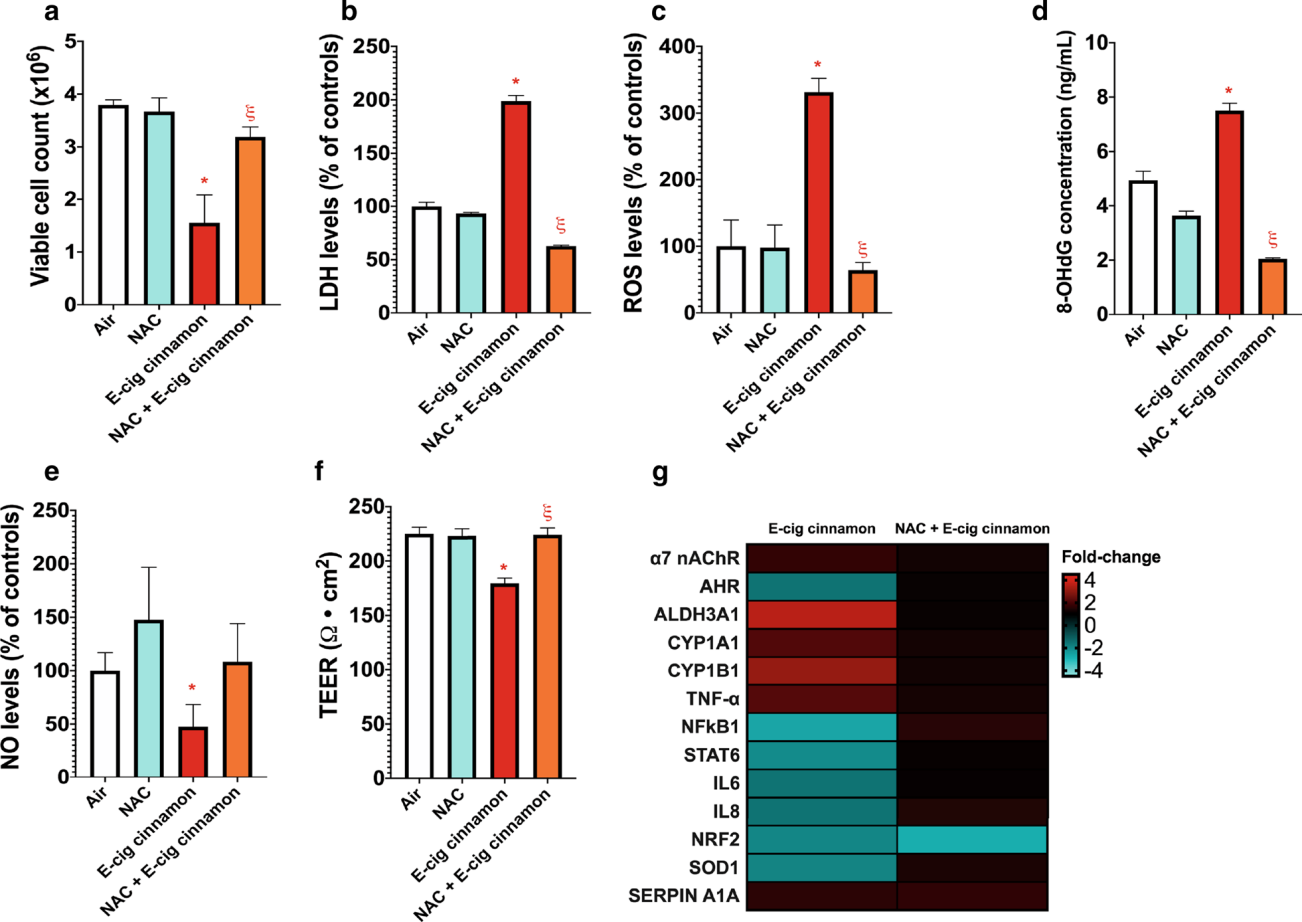
1 Day of cinnamon-flavored e-cig aerosol exposure under sub-ohm conditions-induced oxidative stress-mediated lung cell injury is alleviated by n-acetyl cysteine (NAC) pre-treatment. H292 cells were pre-treated with or without NAC at a concentration of 5 mM for 12 h before exposure to cinnamon-flavored e-cig aerosol for 1 day at the air–liquid interface (ALI). **a** Exposure to cinnamon-flavored e-cig aerosol significantly reduced cellular viability, and NAC pre-treatment prevented this decrease. **b** Levels of extracellular lactate dehydrogenase (LDH) were significantly increased by the cinnamon-flavored e-cig aerosol compared to its respective air control; NAC pre-treatment prevented this elevation. **c** Levels of extracellular reactive oxygen species (ROS) were significantly increased by the cinnamon-flavored e-cig aerosol compared to its respective air control; NAC pre-treatment allowed ROS to remain at control levels. **d** The extracellular protein concentration of 8-OHdG was significantly increased by the cinnamon-flavored e-cig aerosol compared to the air control group, and NAC pre-treatment significantly reduced the concentration. This ELISA was analyzed for cell inserts (n = 3 per group). Each cell insert was further evaluated in duplicate. **e** Levels of extracellular nitric oxide (NO) were significantly reduced by the cinnamon-flavored e-cig aerosol compared to respective air control; and NAC pre-treatment did not significantly change this response. **f** The transepithelial electrical resistance (TEER) values were significantly decreased by the cinnamon-flavored e-cig aerosol compared to its respective air control, and NAC pre-treatment significantly improved the TEER values. For assays (**a**, **c**, **f**), data are from one experiment representative of results from three independent experiments, each performed in triplicate (n = 3 per group). For each cell insert, bioassays were further evaluated in duplicate or triplicate. For all assays (**b–f**) data were normalized to cell count and are presented as mean ± SEM. Comparisons between groups were made with one way ANOVA followed by the Tukey's Multiple Comparison Test. *p < 0.05: significantly different from respective air control; ^ξ^*p* < 0.05: significantly different from e-cig aerosol exposed-cells without NAC pre-treatment. **g** Heatmap of dysregulated genes in H292 cells pre-treated with or without NAC followed by exposure to cinnamon-flavored e-cig aerosol. Data are from H292 cells from distinct experiments. Data are presented as fold-change over the respective air-control group. Fold-changes > ± 1.5 were considered significant. Results from other independent experiments are represented in Additional file 1: Figures S4 and S5

Since ROS levels were elevated in lung cells exposed to either butter- or cinnamon-flavored e-cig aerosols (Fig. 4c), we investigated whether pre-treating lung cells for 12 h with 5 mM of NAC**,** a potent antioxidant and scavenger of free radicals, would help protect lung cells against ROS-mediated injury. Although butter-flavored e-cig aerosol did not alter cell viability (Fig. 5a), this exposure led to an increase in levels of extracellular LDH and ROS, which were prevented in cells pre-treated with NAC (Fig. 5b, c). In contrast, lung cells exposed to cinnamon-flavored e-cig aerosol exhibited a greater than 50% decrease in the numbers of viable cells (Fig. 6a). This was supported by significant increases in the levels of extracellular LDH and ROS (Fig. 6b, c). Cell numbers, LDH and ROS stayed at control levels in NAC-pretreated cells (Fig. 6a–c). In addition, pre-treatment with NAC prevented an increase in 8-OHdG concentration that would otherwise occur only in cinnamon-flavored e-cig aerosol exposed-cells (Fig. 6d). While extracellular NO levels were not significantly decreased in butter-flavored e-cig aerosol exposed-cells when compared to respective air controls, pre-treating the cells with NAC significantly increased the NO levels (> 100%) when compared to NAC untreated e-cig-exposed cells (Fig. 5e). For the cinnamon-flavored e-cig aerosol, NAC pre-treatment did not significantly prevent the decrease in extracellular NO levels (Fig. 6E). In addition, NAC pre-treatment prevented the significant reduction in TEER measurements for both butter- and cinnamon-flavored e-cig aerosol (Figs. 5f, 6f). The NAC pre-treatment had a profound effect on gene expression as well (Figs. 5g, 6g). While only 7 genes were dysregulated by the butter-flavored e-cig aerosol (Fig. 5g), cinnamon-flavored e-cig aerosol exposed-cells exhibited elevated levels of gene dysregulation (Fig. 6g). Of the 13 dysregulated genes in the cinnamon-flavored e-cig aerosol exposed-cells that were not pre-treated with NAC, 7 were down-regulated, while 6 were up-regulated (Fig. 6g). Biotransformation, oxidative stress and inflammation pathway genes predominated (Fig. 6g). NAC pre-treatment prevented the dysregulation of those genes with the exception of *NRF2*, *NF-kB1* and *SERPINA1A* (Fig. 6g). These findings suggest that pre-treatment with NAC may alleviate the oxidative stress-mediated damage to lung cells induced by flavored e-cig aerosols, with NAC being more effective preventing cinnamon-flavored e-cig aerosol lung injury.

### 3 Days of e-cig aerosol exposure under sub-ohm conditions revealed that cinnamon flavor is more cytotoxic and causes oxidative damage to a greater extent than butter-flavored e-cig aerosol

**Fig. 7:**
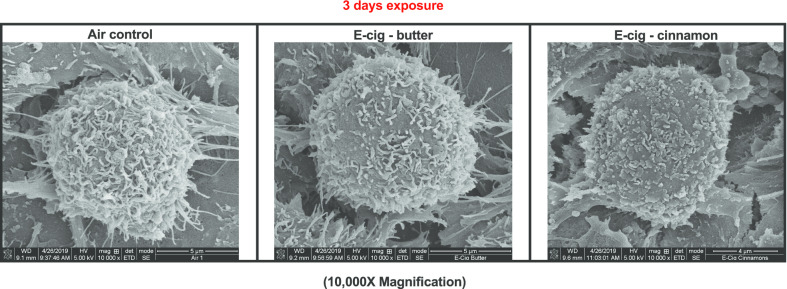
Ciliary abnormalities are observed on H292 cells following 3 days of exposures to e-cig aerosol generated under sub-ohm conditions. Scanning electron microscopy (SEM) images of representative H292 cells exposed to either air, butter- or cinnamon-flavored e-cig aerosols for 3 days at the air–liquid interface (ALI). Representative images for each exposure group. For air-exposed cells: a total of 30 SEM images were taken; for butter-flavored e-cig aerosol-exposed cells: a total of 16 SEM images were taken; for cinnamon-flavored e-cig aerosol-exposed cells: a total of 9 SEM images were taken

**Fig. 8.**
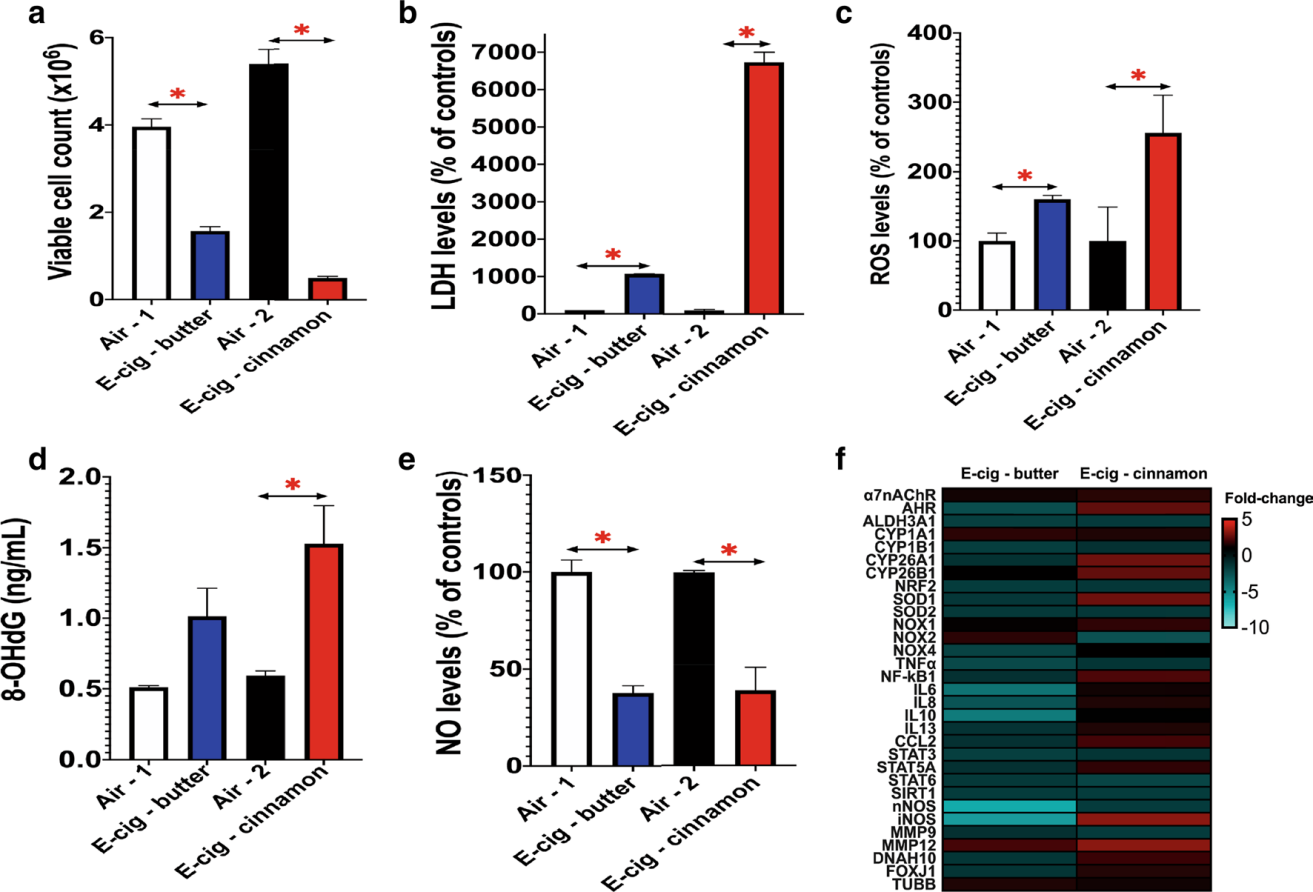
3 Days of e-cig aerosol exposure under sub-ohm conditions (0.15 Ω & 4.8 V) revealed that cinnamon flavor is more cytotoxic and causes oxidative damage to a greater extent than butter flavored-e-cig aerosol. H292 cells were exposed to either butter- or cinnamon-flavored e-cig aerosol at the air–liquid interface (ALI) for 3 days. **a** Numbers of viable cells were significantly decreased by both butter- and cinnamon-flavored e-cig aerosols compared to respective air controls. **b** Levels of extracellular lactate dehydrogenase (LDH) were significantly increased by both butter- and cinnamon-flavored e-cig aerosols compared to respective air controls. **c** Levels of extracellular reactive oxygen species (ROS) were significantly increased by both butter- and cinnamon-flavored e-cig aerosols compared to their respective air controls. **d** The extracellular protein concentration of 8-OHdG was significantly increased by the cinnamon-flavored e-cig aerosol compared to the air control group. This ELISA was analyzed for cell inserts (n = 3 per group), and each cell insert was further evaluated in duplicate. **e** Levels of extracellular nitric oxide (NO) were significantly decreased by both butter- and cinnamon-flavored e-cig aerosols compared to respective air controls. For assays (**a**, **b**, **c**, **e**), data are from one experiment representative of results from three independent experiments, each performed in triplicate (n = 3 per group). For each cell insert, bioassays were further evaluated in duplicate or triplicate. For all assays (**b–e**) data were normalized to cell count and are presented as mean ± SEM. Comparisons between e-cig group and respective air control group were made by the student t-test; *p < 0.05: significantly different from respective air control. **f** Heatmap of the dysregulated genes by the e-cig aerosol exposures. Data are from H292 cells from distinct experiments. Data are presented as fold-change over the respective air-control group (n = 3 per group, each sample run in duplicate). Fold-changes > ± 1.5 were considered significant.Results from other independent experiments are represented in Additional file 1: Figures S6 to S9

Ciliary abnormalities are observed on H292 cells following 3 days of exposures to e-cig aerosol generated under sub-ohm conditions (Fig. 7). Cinnamon-flavored e-cig aerosol exposed-cells appeared visually to be more affected than butter-flavored exposed-cells. This is supported at the molecular level with the up-regulation of *DNAH10* (1.9-fold) (Fig. 8f), a gene that codes for axonemal dyneins, which are microtubule-associated motor protein complexes found in cilia and flagella [51]. Their main function is to generate proteins of respiratory system cilia [52]. In addition, cinnamon-flavored e-cig aerosol also up-regulated the expression of *FOXJ1* (1.6-fold) (Fig. 8f), a protein coding gene involved in ciliogenesis [51, 53]. In contrast, the butter-flavored e-cig aerosol only up-regulated the expression of tubulin beta class I (*TUBB*) by 1.5-fold (Fig. 8f). *TUBB* codes for a major protein component of the ciliary axoneme [54]. This suggests that e-cig aerosol exposures, similarly to cigarette smoke exposures, can affect respiratory cilia, for which alterations are associated with decreased mucociliary clearance [55]. Although the cell-deposited doses were similar between both exposure groups, 33.8 μg/cm^2^ ± 6.5 and 29.5 μg/cm^2^ ± 4.6, for butter- and cinnamon-flavored e-cig aerosols, respectively, the gene expression of *α7nAChR*, measured 24 h after the last e-cig exposure, was significantly increased by 1.6-fold in the cinnamon-flavored e-cig aerosol-exposed cells (Fig. 8f). Only the cinnamon-flavored e-cig aerosol significantly affected cellular viability after 1 day of exposure (Fig. 4a); however, both butter- and cinnamon-flavored e-cig aerosols significantly decreased the number of viable H292 cells following 3 days of exposure (Fig. 8a). Along with the significant reduction in cellular viability, we found significantly elevated levels of extracellular LDH and ROS following exposures to both e-cig aerosols (Fig. 8b, c). The concentration of 8-OHdG, an indicator of oxidative DNA damage, was significantly increased only in the cell culture medium of the cinnamon-flavored e-cig aerosol-exposed-cells (Fig. 8d). The extracellular NO levels were significantly decreased by both e-cig aerosol exposures (Fig. 8e). Gene expression changes were notably greater in the cinnamon-flavored e-cig aerosol-exposed H292 cells than in the butter-flavored-exposed cells (Fig. 8f). In butter-flavored e-cig aerosol-exposed cells, 14 genes, including inflammatory genes (*IL-6*, *IL-8*, *IL-10* and *TNFα*) were down-regulated while only four genes (*CYP1A1*, *NOX2*, *MMP12*, and *TUBB*) were up-regulated (Fig. 8f). Among cinnamon-flavored e-cig aerosol-exposed cells, 16 genes were up-regulated, including genes associated with biotransformation, oxidative stress and inflammation, while 6 genes were down-regulated (Fig. 8f). Overall, as was the case with 1-day exposures under sub-ohm conditions, the effects were more potent and pro-oxidant in cells exposed to cinnamon-flavored e-cig aerosol than to butter-flavored e-cig aerosol.

## Discussion

Multiple factors can influence the physical and chemical characteristics of e-cig aerosols. These factors are associated with the ratios and constituents of the e-liquid formulation, which impact the chemical component of the aerosol, as well as the atomizer’s resistance and voltage applied to the e-cig device, which influence the heating conditions used to aerosolize the e-liquid, while also affecting the physical properties of the aerosol, including particle size. Most importantly, e-cig users inhale these aerosols and control the choice of both the e-liquid and the design features (resistance and voltage) of their third or fourth generation e-cig devices. These are key factors, based on personal e-cig vaping preferences, which can significantly impact the toxicity of the inhaled e-cig aerosol. In the present study, we established the influence of two design characteristics of third-generation tank-style e-cig devices—atomizer resistance and battery voltage—on butter- and cinnamon-flavored e-cig aerosols’ composition (Fig. 1), as well as on their cellular toxicity in vitro (Figs. 2, 3, 4, 5, 6, 7, 8). We found higher concentrations of carbonyls when the butter-flavored e-cig aerosols were produced under sub-ohm vaping conditions (Fig. 1). Additionally, the production of those harmful chemicals may be flavor-specific, since the cinnamon-flavored e-cig aerosols, under the same exposure conditions, did not show elevated levels of carbonyls (Fig. 1). For the butter flavor, the increased levels of carbonyls produced under sub-ohm vaping conditions translated into increased H292 cellular toxicity when compared to regular vaping conditions (Fig. 3). We also found, despite the lower levels of carbonyls, that 1 and 3 days of exposures to cinnamon-flavored e-cig aerosols were more toxic and induced pro-oxidant effects to a greater extent than exposures to butter-flavored e-cig aerosols produced under sub-ohm conditions (Figs. 1, 4, 5, 6, 7, 8). This strongly suggests that the carbonyls present in the e-cig aerosols (Fig. 1) may not be solely responsible for the cellular toxicity (Figs. 4, 5, 6, 7, 8), but that the intrinsic toxicity of the flavoring chemical, such as the well-known deleterious effects of cinnamaldehyde [36, 46–50], may also play a significant role. Overall, we showed (1) that sub-ohm (0.15 Ω) vaping produces more toxic chemicals than regular (1.5 Ω) vaping, and that this can be flavor specific; and (2) that sub-ohm vaping induces detrimental effects to human lung epithelial cells.

Carbonyls are harmful chemicals present in cigarette smoke, some with carcinogenic and mutagenic properties, which are associated with smoking-related lung diseases [56]. Those carbonyls include acetaldehyde, formaldehyde and acrolein. Both acetaldehyde and formaldehyde are irritants to the mucous membranes of the respiratory tract and are classified as probable human carcinogens [17, 23]; while inhaled acrolein is a highly toxic irritant that can affect lung function [17, 23, 57]. Those carbonyls are also found in e-cig aerosols, since they are produced by the thermal degradation of the e-liquid base constituents, PG and VG, following heating and aerosolization processes through an e-cig device [56]. The concentrations, however, of these carbonyls in e-cig aerosols produced under ‘real-life’ human exposure conditions is still conflicting, with some studies showing levels significantly below those found in cigarette smoke, while others demonstrate levels as high or higher than in cigarette smoke [17, 19–23, 56]. The discrepancy of these results is also fueled by the lack of standardized procedures for puffing topography, as well as for the collection and analytical methods used to quantify the carbonyls in the e-cig aerosols. In our study, the aerosols were not produced under dry puff conditions and for the butter flavor showed that the concentration of carbonyls increased when produced under sub-ohm vaping conditions compared to regular vaping conditions (Fig. 1A). This indicates that for butter-flavored e-cig aerosols, carbonyl levels increase when increasing power is applied to the e-cig device, since P = V^2^/R, where P is power in watts, V is voltage in volts, and R is resistance in ohms [24]. These results are in line with another study that used a 50/50 PG/VG (without nicotine) e-liquid and showed increased levels of acetaldehyde, formaldehyde and acrolein produced with atomizers’ resistance of 0.25 vs. 1.5 Ω and 3.5 V applied to the e-cig device [16]. Interestingly, they also found that for the same e-liquid to which 18% of nicotine was added, the levels of acetaldehyde and formaldehyde in the e-cig aerosol were significantly higher when produced with the atomizer of 1.5 Ω compared to 0.25 Ω [16]. In addition, overall, the levels of carbonyls produced with the e-liquid containing no nicotine were substantially higher than those generated with the 18% nicotine-containing e-liquid [16]. Other studies [19, 28] also demonstrated changes in the chemical and physical properties of the e-cig aerosols when nicotine is present in the e-liquids. In our study, under the same exposure conditions as for the butter flavor, the cinnamon-flavored e-cig aerosols produced much lower levels of carbonyls (Fig. 1b). The effect of the presence of flavoring chemicals in e-liquids on the concentration of carbonyls found in e-cig aerosols have not been extensively studied; however, it was previously reported that the concentrations of carbonyls in e-cig aerosols varied between flavored e-liquids, including bubble gum and gummy bear flavors [58]. It is thought that the thermal degradation of flavoring chemicals may interact with that of PG and VG, and this can affect the overall levels of carbonyls produced in the e-cig aerosols [58]. This suggests that the chemical composition of the e-liquid (PG, VG, nicotine and flavors) may have a significant effect on the emission of carbonyls in the e-cig aerosols. This supports our data (Fig. 1), which show that the production of high levels of carbonyls produced under sub-ohm vaping conditions is flavor specific.

Our exposure system composed of a third-generation e-cig device connected to an ALI system can provide exposure conditions at an in vitro dose range similar to other studies (0.2–182 μg/cm^2^) [13–15, 17, 23, 33], that used ALI for e-cig aerosol exposures. Those levels are considered equivalent to values of human pulmonary deposition fraction [13–15, 17, 23, 33]. We showed that for butter-flavored e-cig aerosols, sub-ohm (0.15 Ω) vaping produced more toxic chemicals than vaping with an atomizer of 1.5 Ω (Fig. 1), and this was translated into increased cellular toxicity, as evidenced by decreased cell viability and altered expression of key genes associated with oxidative stress, airway remodeling, and inflammation (Fig. 3). These data suggest that the increased cytotoxicity observed with sub-ohm vaping may be associated with the higher carbonyl content of the e-cig aerosol (Figs. 1, 3). In line with our results, it was previously reported in an ALI exposure system that the viability of human lung carcinoma cells (H1299) was significantly decreased when exposed to a 50/50 PG/VG, nicotine-free raspberry-flavored e-cig aerosol produced under sub-ohm conditions (0.25 Ω atomizer and 3.5 V) compared to both control cells and cells exposed under regular vaping conditions (1.5 Ω atomizer and 3.5 V) [16]. In addition, that study also showed that cell viability was inversely correlated with carbonyl levels and ROS generation from the e-cig aerosols [16]. The detrimental effects of sub-ohm versus regular vaping were also investigated in vivo [59]. Rats exposed to 50/50 PG/VG, nicotine-free fruit-flavored e-cig aerosols, produced under sub-ohm conditions for 28 days, exhibited dysregulation of antioxidant responses, up-regulation of biotransformation genes, including *Cyp1a1* and *Cyp2E1*, increased ROS levels in lungs, and altered hematological profile when compared to controls rats or rats exposed to e-cig aerosol produced under regular vaping conditions [59]. Moreover, by using SEM and transmission electron microscopy (TEM) images the study also demonstrated lung and trachea tissue damage, including large areas of lung airflow collapse that were more pronounced when rats were exposed to the e-cig aerosols produced under sub-ohm conditions [59]. Taken together, these two studies and our results strongly indicate that sub-ohm vaping can cause harmful effects to lung cells and the respiratory tract, which may be worse than pulmonary effects due to regular vaping. This also suggests that the use of e-cig devices with sub-ohm atomizers (< 0.5 Ω) should be restricted, as it may increase the risks of adverse pulmonary responses.

Butter flavoring chemicals include diacetyl, acetyl propionyl and acetoin [60]. Diacetyl is a well-known inhaled toxicant that can produce irreversible lung damage, including bronchiolitis obliterans [61, 62]. The butter-flavored e-liquid that we used, according to the manufacturer, was diacetyl-free. It was reported that acetyl propionyl and acetoin can be used as a substitute for diacetyl, based on their similar chemical structures [60, 62]. As demonstrated in rodents, both of these compounds may induce comparable lung effects to diacetyl; however, with acetoin showing significantly milder effects [61, 62]. Although butter-flavored aerosols contained higher levels of carbonyls than their cinnamon counterparts (Fig. 1), we found that 1 and 3 days of exposure to cinnamon-flavored e-cig aerosols are more toxic than exposures to butter-flavored aerosols (Figs. 4, 7, 8). It was previously reported that the median lethal concentration (LC_50_) (% volume/volume) for butter-flavored e-liquids ranged between 2.1 and 3.0, while that for cinnamon roll e-liquid was 0.7 [63]. This suggests that the intrinsic toxicity of cinnamon-related e-liquids may be higher than that of buttery based e-liquids. Therefore, aside from the carbonyls produced (Fig. 1), the enhanced toxicity of the cinnamon-flavored e-cig aerosol (Figs. 4, 7, 8) may be due to cinnamaldehyde, the primary flavoring chemical of cinnamon-based e-liquids, which was shown to be highly toxic in both in vitro and in vivo models [36, 46–50, 64]. For instance, using a zebrafish model it was shown that the presence of cinnamaldehyde in various e-liquids, including in bubble gum flavored-e-liquids, was mainly responsible for the harmful developmental effects observed [65]. Another study in osteoblast cells showed that cinnamon-flavored e-liquid-augmented cytotoxicity was related to oxidative stress, as evidence by increased ROS production [66]. In both in vitro and in vivo models, Lerner et al*.* [67] showed that e-cig aerosols’ toxicity, including that of cinnamon flavors, was associated with oxidative stress. NAC is an antioxidant shown to provide protection against ROS-mediated lung injury in vitro and in vivo [68, 69]. We showed that pre-treating the cells with NAC prevented butter- and cinnamon-flavored e-cig aerosols-mediated cellular toxicity by attenuating LDH release, ROS production and improving TEER levels (Figs. 5, 6). In addition, NAC circumvented the decrease in viable cell number and oxidative DNA damage induced by the cinnamon-flavored e-cig aerosol (Fig. 6). Accordingly, our data suggest that cinnamon-flavored aerosols may be more pro-oxidant than butter-flavored e-cig aerosols since only the cinnamon-flavored e-cig aerosols significantly increased 8-OHdG protein concentrations, a marker of oxidative DNA damage, following 1 and 3 days of exposure (Figs. 4, 8). These results are in line with other studies that showed oxidative DNA lesions in human oral and bronchial cells exposed to e-cig aerosols or extracts [70, 71]. In our study, the pro-oxidant effect of the cinnamon-flavored e-cig aerosol was also seen at the molecular level, with the dysregulation of antioxidant response-related genes, including *SOD1* and *NOX1/2*, at both time-points, in addition to *NRF2* and *DUOX2* following 1 day of exposure (Figs. 4, 8). Pre-treating the cells with NAC allowed most genes dysregulated by the cinnamon-flavored e-cig aerosol to remain at baseline levels (Fig. 6). Overall, these data point towards a major role of oxidative stress in cinnamon-flavored e-cig aerosol-mediated H292 cellular toxicity. It was previously reported that out of 49 flavored e-liquids tested, the ‘*subtle cinnamon’*-flavored e-liquid ranked in the top 5 for the production of free radicals found in the e-cig aerosol with a significant 105% increase compared to the base PG/VG aerosol [72]. This supports the notion that cinnamon-flavored e-cig aerosol may induce greater oxidative damage to lung cells due to the increased presence of free radicals.

Overall, our data suggest that e-cig aerosol toxicity may be flavor-specific (Figs. 1, 2, 3, 4, 5, 6, 7, 8). Indeed, in terms of cellular toxicity (Figs. 4, 7, 8), we showed that exposures to e-cig aerosols at the ALI under sub-ohm conditions are toxic to lung epithelial cells, with cinnamon-flavored e-cig aerosols producing more adverse effects than butter-flavored e-cig aerosols. This is of significant importance to public health, since cinnamaldehyde and diacetyl, which, as we (Figs. 4, 5, 6, 7, 8) and others have shown, are cytotoxic in vitro and in vivo [36, 46–50, 64], and have been identified as high-priority flavoring chemicals for evaluation of respiratory hazard [73], are very popular in e-liquids. Diacetyl was detected in 76% of 51 commercially available e-liquids tested [74], whereas, in addition to cinnamon-flavored e-liquids, cinnamaldehyde was detected in 44% of 27 non-cinnamon labelled e-liquids tested [49]. This suggests that buttery- and cinnamon-related flavoring chemicals are present in a wide variety of e-liquids on the market, without users necessarily realizing this; thus, exposures of e-cig users to potentially harmful e-cig aerosols may be widespread.

Mucociliary clearance is a major primary defense mechanism of the respiratory tract [75]. This physical barrier includes airway mucus and motile cilia present at the surface of ciliated cells of the bronchial epithelium [75]. In in vitro models, the presence of cilia at the surface of bronchial epithelial cells support differentiation of the cells at the ALI (Fig. 2). Qualitative observations of SEM images suggest that 3 days of exposure to e-cig aerosols may lead to shorter cilia at the surface of the cells (Fig. 7). As visualized by SEM, the cinnamon-flavored e-cig aerosol-exposed cells appeared to have fewer and shorter cilia compared to both butter-flavored e-cig aerosol-exposed cells and controls (Fig. 7). The ciliary abnormalities of cinnamon-flavored e-cig aerosol-exposed cells were supported by the up-regulation of *DNAH10* (1.9-fold) and *FOXJ1* (1.6-fold) (Fig. 8f), two protein-coding genes that play significant roles in ciliogenesis, cilia assembly and movement [51, 53]. This increase in gene expression may suggest that the cells are seeking to increase the synthesis of these proteins to help replace the cilia barrier. Moses et al*.* [51] reported in human primary bronchial epithelial cells that 1 day of ALI exposure to menthol and tobacco-flavored e-cig aerosols produced with a disposable Blue-brand e-cig device, resulted in down-regulation of *DNAH10* and *FOXJ1*. In our ALI exposure model, we showed in H292 cells that the gene expression of DNAH10 and *FOXJ1* was up-regulated following 3 days of exposure to the cinnamon-flavored e-cig aerosol, and that *TUBB* gene expression was up-regulated after 3 days of exposure to butter-flavored e-cig aerosol, produced by a third-generation e-cig device (Fig. 8f). Despite the numerous different experimental conditions used in these 2 studies, our results do not necessarily conflict with those of Moses et al*.* [51], as they may reflect differences in exposure duration temporal profiles (1 vs. 3 days) of cilia-related gene expression following e-cig aerosol exposure; and initial down-regulation, which could lead to ciliary abnormalities (fewer and shorter cilia), followed by up-regulation to replenish the cell surface. An important takeaway from these two studies is that short-term e-cig aerosol exposures dysregulated the expression of cilia-related genes, which are essential for healthy airway physiology. Just as it was demonstrated previously with cigarette smoke exposures and other inhaled pollutants [76, 77], recently, it was shown that e-cig aerosol exposures affected cilia and mucociliary clearance mechanisms of the respiratory epithelium [59, 75, 78]. In vivo, rats exposed to e-cig aerosols produced under either sub-ohm or regular vaping conditions showed trachea tissue epithelium detachment and loss of cilia, as visualized by TEM, with sub-ohm vaping causing more pronounced damage [59]. In both human cells and in a sheep in vivo model, Chung et al. [78] demonstrated that exposures to e-cig aerosol-induced airway mucociliary dysfunction by increasing the mucus viscosity and reducing the mucus velocity. Furthermore, it was previously shown that human bronchial epithelial cells exposed to cinnamaldehyde e-cig aerosols displayed transient suppression of ciliary mobility, as evidenced by reduced ciliary beat frequency [75]. Moreover, at the molecular level, it was also demonstrated that e-cig aerosol exposures dysregulate the expression of genes associated with airway cilia in human bronchial epithelial cells [51]. Taken together, our results and those of others have shown that e-cig aerosol exposures can affect cilia length and mucociliary function, which are associated with decreased mucociliary clearance. It is well-established that decreased mucociliary clearance can increase the risk of respiratory infections and injury to the epithelium [75–77]. Thus, the weight of recent results, including those we present here, suggest that e-cig aerosol exposures may pose a threat to lung health.

This study includes some potential limitations. First, the chemical profiles of the e-cig aerosols generated under 9 different heating conditions for the 2 e-liquid flavors analyzed (butter and cinnamon; Fig. 1) represent a one-time snapshot providing insights into the harmful chemicals present in these aerosols and their relative levels. Very few studies [16] have exhaustively assessed the characterization of the e-cig exposures, including the analysis of the e-liquid components as well as the e-cig aerosol chemical profiles for nicotine, PG, VG and carbonyls, in addition to a thorough evaluation of the cellular toxicity. The study presented here includes all of those analyses, in addition to SEM images of the cells cultured at the ALI following exposures to e-cig aerosols. This enables a better understanding of the exposure-effect continuum as it pertains to the correlation between the e-cig aerosol characteristics and the responses of cells at the morphological, cellular and molecular levels. Second, our in vitro ALI exposure model did not use primary human bronchial epithelial cells. Validation of some of the key results with primary human bronchial cultures would greatly strengthen and improve the translational aspect of this study. Primary cells, however, differ from batch-to-batch and are more challenging to maintain than cell lines. This represents a significant disadvantage when conducting high throughput screening and toxicological testing [79]. Published studies have shown consistent evidence that different types of human bronchial cell lines (BEAS-2B; Calu-3; HFL-1; H292) and primary human bronchial epithelial cells display different cellular toxicity profiles following in vitro exposures to tobacco-related products or their constituents [48; 80]. For instance, 7 different e-liquid flavoring chemicals induced significant IL-8 release from BEAS-2B and HFL-1 cells, while under the same exposure conditions, no significant IL-8 induction was observed in H292 cells [48]. In another study, while IL-8 concentration was significantly increased in Calu-3 cells exposed to e-cig aerosols at the ALI when compared to control cells, H292 cells and primary human bronchial epithelial cells did not significantly augment the synthesis of IL-8 from baseline control levels [80]. These results have at least two implications: 1) H292 cells and primary human lung epithelial cells may exhibit similar IL-8 release cellular responses after exposure to e-cig aerosols at the ALI; and 2) that in an ALI exposure model, H292 cells, although a cancer cell-line, may be a relevant surrogate for primary human bronchial epithelial cells, which are more complex and difficult to use reliably in large scale and long-term experimental ALI context. Third, since the e-cig aerosol exposures were conducted for either 1 day or 3 consecutive days, the data are representative of short-term exposure effects rather than of responses to prolonged exposure to e-cig aerosols. Therefore, this study should not be used to infer the long-term effects associated with the chronic use of e-cig devices. Finally, as in vitro exposures were conducted on a human lung cell line rather than on primary human lung cells, this may negatively influence the translational impact and thus the generalization potential of the data presented here.

## Conclusion

In the global context of public health, our results suggest that the use of e-cig devices with sub-ohm atomizers (< 0.5 Ω) should be prevented, as sub-ohm vaping induced detrimental effects on lung cells due to cytotoxicity, enhanced oxidative stress, low levels of nitric oxide, diminished TEER, and altered expression of key genes associated with biotransformation, oxidative stress, and inflammation. In addition, e-cig aerosols induce cellular toxicity via mechanisms potentially associated with oxidative stress. Our results also suggest that e-cig aerosol toxicity may be flavor-specific. Overall, our data underline that e-cigs may not be a “safe” alternative to conventional cigarettes. Taken together, our results could help policymakers take the necessary steps to prevent the use or manufacturing of sub-ohm (i.e. 0.15 Ω) atomizers. More research is needed to determine the longer-term effects of e-cig aerosol exposures, using human primary bronchial epithelial cells, as well as the in vivo pulmonary responses.

## Supplementary information

**Additional file 1: Table S1.** Primers used for qRT-PCR analysis of human genes. **Figure S1.** 3 days of butter-flavored e-cig aerosol exposure under sub-ohm conditions decrease viable cell numbers (trials #2 and 3). A) Numbers of viable cells were significantly decreased by the e-cig aerosol produced under sub-ohm vaping conditions (trial #2). B) Numbers of viable cells were decreased by the e-cig aerosol produced under sub-ohm vaping conditions (trial #3). Data are presented as mean ± SEM (n = 3 cell inserts per group). Comparisons between groups were made by the student t-test; *p < 0.05: significantly different from the other group. **Figure S2.** 1 day of butter-flavored e-cig aerosol exposure under sub-ohm conditions (0.15 Ω & 4.8 V) affects the integrity of H292 cells’ tight junctions (trial #2). H292 cells were exposed to butter-flavored e-cig aerosol at the air–liquid interface (ALI) for 1 day. A) Numbers of viable cells. B) Levels of extracellular lactate dehydrogenase (LDH). C) Levels of extracellular reactive oxygen species (ROS) were significantly increased by butter-flavored e-cig aerosol compared to the air control group. D) Levels of extracellular nitric oxide (NO). E) Exposure of cells to NAC pre-treatment and NAC pre-treatment plus butter-flavored e-cig aerosol had no significant effect on cellular viability. F) The transepithelial electrical resistance (TEER) values were significantly decreased by butter-flavored e-cig aerosols compared to the respective air control group. A-F: Data are presented as mean ± SEM (n = 3 cell inserts per group). For each cell insert, bioassays were further evaluated in duplicate or triplicate. For assays B – D, data were normalized to cell count. Comparisons between the e-cig group and the air control group were made by the student t-test; *p < 0.05: significantly different from air control; ^ξ^*p* < 0.05: significantly different from e-cig aerosol exposed-cells without NAC pre-treatment. **Figure S3.** 1 day of butter-flavored e-cig aerosol exposure under sub-ohm conditions (0.15 Ω & 4.8 V) affects the integrity of H292 cells’ tight junctions (trial #3). H292 cells were exposed to butter-flavored e-cig aerosol at the air–liquid interface (ALI) for 1 day. A) Numbers of viable cells. B) Levels of extracellular lactate dehydrogenase (LDH). C) Levels of extracellular reactive oxygen species (ROS). D) Levels of extracellular nitric oxide (NO). E) Exposure of cells to NAC pre-treatment and NAC pre-treatment plus butter-flavored e-cig aerosol had no significant effect on cellular viability. F) The transepithelial electrical resistance (TEER) values were significantly decreased by butter-flavored e-cig aerosols compared to the respective air control group. A-F: Data are presented as mean ± SEM (n = 3 cell inserts per group). For each cell insert, bioassays were further evaluated in duplicate or triplicate. For assays B – D, data were normalized to cell count. Comparisons between the e-cig group and the air control group were made by the student t-test; *p < 0.05: significantly different from air control; ^ξ^*p* < 0.05: significantly different from e-cig aerosol exposed-cells without NAC pre-treatment. **Figure S4.** 1 day of cinnamon-flavored e-cig aerosol exposure under sub-ohm conditions (0.15 Ω & 4.8 V) decreases cell viability and affects the integrity of H292 cells’ tight junctions (trial #2). H292 cells were exposed to cinnamon-flavored e-cig aerosol at the air–liquid interface (ALI) for 1 day. A) Numbers of viable cells were significantly decreased by the cinnamon-flavored e-cig aerosol compared to air control. B) Levels of extracellular lactate dehydrogenase (LDH) were significantly increased by cinnamon-flavored e-cig aerosols compared to the air control group. C) Levels of extracellular reactive oxygen species (ROS) were significantly increased by cinnamon-flavored e-cig aerosols compared to the air control group. D) Levels of extracellular nitric oxide (NO). E) Exposure of cells to NAC pre-treatment and NAC pre-treatment plus cinnamon-flavored e-cig aerosol had no significant effect on cellular viability. F) The transepithelial electrical resistance (TEER) values were significantly decreased by cinnamon-flavored e-cig aerosols compared to the air control group. A-F: Data are presented as mean ± SEM (n = 3 cell inserts per group). For each cell insert, bioassays were further evaluated in duplicate or triplicate. For assays B—D data were normalized to cell count. Comparisons between the e-cig group and the air control group were made by the student t-test; *p < 0.05: significantly different from air control; ^ξ^*p* < 0.05: significantly different from e-cig aerosol exposed-cells without NAC pre-treatment. **Figure S5.** 1 day of cinnamon-flavored e-cig aerosol exposure under sub-ohm conditions (0.15 Ω & 4.8 V) decreases cell viability and affects the integrity of H292 cells’ tight junctions (trial #3). H292 cells were exposed to cinnamon-flavored e-cig aerosol at the air–liquid interface (ALI) for 1 day. A) Numbers of viable cells. B) Levels of extracellular lactate dehydrogenase (LDH) were significantly increased by cinnamon-flavored e-cig aerosols compared to the air control group. C) Levels of extracellular reactive oxygen species (ROS). D) Levels of extracellular nitric oxide (NO). E) Exposure of cells to NAC pre-treatment and NAC pre-treatment plus cinnamon-flavored e-cig aerosol had no significant effect on cellular viability. F) The transepithelial electrical resistance (TEER) values were significantly decreased by cinnamon-flavored e-cig aerosols compared to the air control group. A-F: Data are presented as mean ± SEM (n = 3 cell inserts per group). For each cell insert, bioassays were further evaluated in duplicate or triplicate. For assays B – D, data were normalized to cell count. Comparisons between the e-cig group and the air control group were made by the student t-test; *p < 0.05: significantly different from air control. **Figure S6.** 3 days of butter-flavored e-cig aerosol exposure under sub-ohm conditions (0.15 Ω & 4.8 V) is cytotoxic and causes oxidative damage to H292 cells (trial #2). H292 cells were exposed to butter-flavored e-cig aerosol at the air–liquid interface (ALI) for 3 days. A) Numbers of viable cells were significantly decreased by butter-flavored e-cig aerosol compared to air controls. B) Levels of extracellular lactate dehydrogenase (LDH) were significantly increased by butter-flavored e-cig aerosol compared to air controls. C) Levels of extracellular reactive oxygen species (ROS) were significantly increased by butter-flavored e-cig aerosol compared to air controls. D) Levels of extracellular nitric oxide (NO) were significantly decreased by butter-flavored e-cig aerosol compared to air controls. A-D: Data are presented as mean ± SEM (n = 3 cell inserts per group). For each cell insert, bioassays were further evaluated in duplicate or triplicate. For assays B – D, data were normalized to cell count. Comparisons between the e-cig group and the air control group were made by the student t-test; *p < 0.05: significantly different from air control. **Figure S7.** 3 days of butter-flavored e-cig aerosol exposure under sub-ohm conditions (0.15 Ω & 4.8 V) is cytotoxic and causes oxidative damage to H292 cells (trial #3). H292 cells were exposed to butter-flavored e-cig aerosol at the air–liquid interface (ALI) for 3 days. A) Numbers of viable cells were significantly decreased by butter-flavored e-cig aerosol compared to air controls. B) Levels of extracellular lactate dehydrogenase (LDH) were significantly increased by butter-flavored e-cig aerosol compared to air controls. C) Levels of extracellular reactive oxygen species (ROS) were significantly increased by butter-flavored e-cig aerosol compared to air controls. D) Levels of extracellular nitric oxide (NO). A-D: Data are presented as mean ± SEM (n = 3 cell inserts per group). For each cell insert, bioassays were further evaluated in duplicate or triplicate. For assays B—D, data were normalized to cell count. Comparisons between the e-cig group and the air control group were made by the student t-test; *p < 0.05: significantly different from air control. **Figure S8.** 3 days of cinnamon-flavored e-cig aerosol exposure under sub-ohm conditions (0.15 Ω & 4.8 V) is cytotoxic and causes oxidative damage to H292 cells (trial #2). H292 cells were exposed to cinnamon-flavored e-cig aerosol at the air–liquid interface (ALI) for 3 days. A) Numbers of viable cells were significantly decreased by cinnamon-flavored e-cig aerosol compared to air controls. B) Levels of extracellular lactate dehydrogenase (LDH) were significantly increased by cinnamon-flavored e-cig aerosol compared to air controls. C) Levels of extracellular reactive oxygen species (ROS) were significantly increased by cinnamon-flavored e-cig aerosol compared to air controls. D) Levels of extracellular nitric oxide (NO) were significantly decreased by cinnamon-flavored e-cig aerosol compared to air controls. A-D: Data are presented as mean ± SEM (n = 3 cell inserts per group). For each cell insert, bioassays were further evaluated in duplicate or triplicate. For assays B—D, data were normalized to cell count. Comparisons between the e-cig group and the air control group were made by the student t-test; *p < 0.05: significantly different from air control. **Figure S9.** 3 days of cinnamon-flavored e-cig aerosol exposure under sub-ohm conditions (0.15 Ω & 4.8 V) is cytotoxic and causes oxidative damage to H292 cells (trial #3). H292 cells were exposed to cinnamon-flavored e-cig aerosol at the air–liquid interface (ALI) for 3 days. A) Numbers of viable cells. B) Levels of extracellular lactate dehydrogenase (LDH) were significantly increased by cinnamon-flavored e-cig aerosol compared to air controls. C) Levels of extracellular reactive oxygen species (ROS) were significantly increased by cinnamon-flavored e-cig aerosol compared to air controls. D) Levels of extracellular nitric oxide (NO). A-D: Data are presented as mean ± SEM (n = 3 cell inserts per group). For each cell insert, bioassays were further evaluated in duplicate or triplicate. For assays B—D, data were normalized to cell count. Comparisons between the e-cig group and the air control group were made by the student t-test; *p < 0.05: significantly different from air control.

## Data Availability

The datasets analyzed during the current study are available from the corresponding author on reasonable request.

## Abbreviations

8-OHdG: 8-Hydroxy**-**2**-**deoxyguanosine
ALI: Air–liquid interface
e-cig: Electronic-cigarette
ENDS: Electronic nicotine delivery system
EVALI: E-cigarette or vaping associated lung injury
GC: Gas chromatography
GRAS: Generally recognized as safe
HPLC: High performance liquid chromatography
LC: Liquid chromatography
LC_50_: Median lethal concentration
LDH: Lactate dehydrogenase
NAC: N-acetyl cysteine
NO: Nitric oxide
OS: Oxidative stress
PG: Propylene glycol
QCM: Quartz microbalance
ROS: Reactive oxygen species
SEM: Scanning electron microscopy
SEM: Standard error of the mean
TEER: Transepithelial electrical resistance
TEM: Transmission electron microscopy
VG: Vegetable glygerin

## References

[1] Centers for Disease Control and Prevention (CDC). Trends in current cigarette smoking among high school students and adults, United States, 1965–2014. https://www.cdc.gov/tobacco/data_statistics/tables/trends/cig_smoking/index.htm. Accessed 28 Jul 2017.

[2] Schoenborn CA, and Gindi RM. Electronic cigarette use among adults: United States, 2014. NCHS Data Brief No. 217, October 2015. US Department of Health and Human Services. Centers for Disease Control and Prevention. National Center for Health Statistics.

[3] Wang T, Asman K, Gentzke AS, Cullen KA, Holder-Hayes E, Reyes-Guzman C (2018). Tobacco product use among adults—United States, 2017. MMWR Morb Mortal Wkly.

[4] Centers for Disease and Prevention (CDC). Fast facts and fact sheets. Youth and tobacco use. https://www.cdc.gov/tobacco/data_statistics/fact_sheets/youth_data/tobacco_use/index.htm#references. Accessed 20 Feb 2020.

[5] Canistro D, Vivarelli F, Cirillo S, Babot MC, Buschini A, Lazzaretti M (2017). E-cigarettes induce toxicological effects that can raise the cancer risk. Sci Rep.

[6] Sleiman M, Logue JM, Montesinos VN, Russell ML, Litter MI, Gundel LA (2016). Emissions from electronic cigarettes: key parameters affecting the release of harmful chemicals. Environ Sci Technol.

[7] Cheng T (2014). Chemical evaluation of electronic cigarettes. Tob Control..

[8] Zhu SH, Sun JY, Bonnevie E, Cummins SE, Gamst A, Yin L (2014). Four hundred and sixty brands of e-cigarettes and counting: implications for product regulation. Tob Control..

[9] Centers for Disease and Prevention (CDC). Basic Information. Electronic-cigarette. Outbreak of lung injury associated with the use of e-cigarette, or vaping, products. https://www.cdc.gov/tobacco/basic_information/e-cigarettes/severe-lung-disease.html. Accessed on 20 Feb 2020.

[10] Vardavas CI, Anagnostopoulos N, Kougias M, Evangelopoulou V, Connolly GN, Behrakis PK (2012). Short-term pulmonary effects of using an electronic cigarette: impact on respiratory flow resistance, impedance, and exhaled nitric oxide. Chest.

[11] Alzahrani T, Pena I, Temesgen N, Glantz SA (2018). Association between electronic cigarette use and myocardial infarction. Am J Prev Med.

[12] Manigrasso M, Buonanno G, Fuoco FC, Stabile L, Avino P (2015). Aerosol deposition doses in the human respiratory tree of electronic cigarette smokers. Environ Pollut.

[13] Scheffler S, Dieken H, Krischenowski O, Forster C, Branscheid D, Aufderheide M (2015). Evaluation of e-cigarette liquid vapor and mainstream cigarette smoke after direct exposure of primary human bronchial epithelial cells. Int J Environ Res Public Health.

[14] Leigh NJ, Lawton RI, Hershberger PA, Goniewicz ML (2016). Flavourings significantly affect inhalation toxicity of aerosol generated from electronic nicotine delivery systems (ENDS). Tob Control..

[15] Garcia-Arcos I, Geraghty P, Baumlin N, Campos M, Dabo AJ, Jundi B (2016). Chronic electronic cigarette exposure in mice induces features of COPD in a nicotine-dependent manner. Thorax.

[16] Cirillo S, Urena JF, Lambert JD, Vivarelli F, Canistro D, Paolini M (2019). Impact of electronic cigarette heating coil resistance on the production of reactive carbonyls, reactive oxygen species and induction of cytotoxicity in human lung cancer cells in vitro. Regul Toxicol Pharmacol.

[17] Geiss O, Bianchi I, Barrero-Moreno J (2016). Correlation of volatile carbonyl yields emitted by e-cigarettes with the temperature of the heating coil and the perceived sensorial quality of the generated vapours. Int J Hyg Environ Health.

[18] Dunbar ZR, Das A, O’Connor RJ, Goniewicz ML, Wei B, Travers MJ (2018). Brief report: lead levels in selected electronic cigarettes from canada and the United States. Int J Environ Res Public Health.

[19] Kosmider L, Sobczak A, Fik M, Knysak J, Zaciera M, Kurek J (2014). Carbonyl compounds in electronic cigarette vapors: effects of nicotine solvent and battery output voltage. Nicotine Tob Res.

[20] Godish T (1989). Formaldehyde exposures from tobacco smoke: a review. Am J Public Health.

[21] Goniewicz ML, Knysak J, Gawron M, Kosmider L, Sobczak A, Kurek J (2014). Levels of selected carcinogens and toxicants in vapour from electronic cigarettes. Tob Control.

[22] Tayyarah R, Long GA (2014). Comparison of select analytes in aerosol from e-cigarettes with smoke from conventional cigarettes and with ambient air. Regul Toxicol Pharmacol.

[23] Geiss O, Bianchi I, Barahona F, Barrero-Moreno J (2015). Characterization of mainstream and passive vapours emmited by selected electronic cigarettes. Int J Hyg Environ Health.

[24] Talih S, Salman R, Karaoghlanian N, El-Hellani A, Saliba N, Eissenberg T (2017). "Juice monsters": sub-ohm vaping and toxic volatile aldehyde emissions. Chem Res Toxicol.

[25] Chaumont M, de Becker B, Zaher W, Culie A, Deprez G, Melot C (2018). Differential effects of e-cigarette on microvascular endothelial function, arterial stiffness and oxidative stress: a randomized crossover trial. Sci Rep..

[26] Smets J, Baeyens F, Chaumont M, Adriaens K, Van Gucht D (2019). When less is more: vaping low-nicotine vs high-nicotine e-liquid is compensated by increased wattage and higher liquid consumption. Int. J Environ Res Public Health..

[27] Kreiss K, Gomaa A, Kullman G, Fedan K, Simoes EJ, Enright PL (2002). Clinical bronchiolitis obliterans in workers at a microwave-popcorn plant. N Engl J Med.

[28] Noël A, Verret CM, Hasan F, Lomnicki S, Morse J, Robichaud A (2018). Generation of electronic cigarette aerosol by a third-generation machine-vaping device: application to toxicological studies. J Vis Exp..

[29] Havel CM, Benowitz NL, Jacob P, St-Helen G (2017). Electronic cigarette vaping machine for the characterization of aerosol delivery and composition. Nicotine Tob Res.

[30] Centre de Cooperation pour les Recherches Scientifiques Relative au Tabac (CORESTA). CORESTA Recommended Method No81. Routine analytical machine for e-cigarette aerosol generation and collection—definitions and standard conditions. June 2015.

[31] Flora JW, Wilkinson CT, Wilkinson JW, Lipowicz PJ, Skapars JA, Anderson A (2017). Method for the determination of carbonyl compounds in e-cigarette aerosols. J Chromatogr Sci.

[32] Azzopardi D, Patel K, Jaunky T, Santopietro S, Camacho OM, McAughey J (2016). Electronic cigarette aerosol induces significantly less cytotoxicity than tobacco smoke. Toxicol Mech Methods.

[33] Taylor M, Carr T, Oke O, Jaunky T, Breheny D, Lowe F (2016). E-cigarette aerosols induce lower oxidative stress in vitro when compared to tobacco smoke. Toxicol Mech Methods.

[34] Ghio AJ, Dailey LA, Soukup JM, Stonehuerner J, Richards JH, Devlin RB (2013). Growth of human bronchial epithelial cells at an air-liquid interface alters the response to particle exposure. Part Fibre Toxicol.

[35] Danahay H, Atherton H, Jones G, Bridges RJ, Poll CT (2002). Ilterleukin-13 induces a hypersecretory ion transport phenotype in human bronchial epithelial cells. Am J Phisiol Lung Cell Mol Physiol.

[36] Noël A, Hansen S, Zaman A, Perveen Z, Pinkston R, Hossain E (2020). In utero exposures to electronic-cigarette aerosols impair the Wnt signaling during mouse lung development. Am J Physiol Lung Cell Mol Physiol.

[37] Murphy G, Rouse RL, Polk WW, Henk WG, Barker SA, Boudreaux MJ (2008). Combustion-derived hydrocarbons localize to lipid droplets in respiratory cells. Am J Respir Cell Mol Biol.

[38] Lesur I, Textoris J, Loriod B, Courbon C, Garcia S, Leone M (2010). Gene expression profiles characterize inflammation stages in the acute lung injury in mice. PLoS ONE.

[39] Maus ADJ, Pereira EFR, Karachunski PI, Horton RM, Navaneetham D, Macklin K (1998). Human and rodent bronchial epithelial cells express functional nicotinic acetylcholine receptors. Mol Pharmacol.

[40] Chan FK, Moriwaki K, De Rosa MJ (2013). Detection of necrosis by release of lactate dehydrogenase activity. Methods Mol Biol.

[41] Ghasemi A, Khanzadeh T, Zadi HM, Khorrami A, Jahanban EA, Ghavipanjeh S (2018). Evaluation of BAX and BCL-2 gene expression and apoptosis induction in acute lymphoblastic leukemia cell line CCRFCEM after high-dose prednisolone treatment. Asian Pac J Cancer Prev.

[42] Hata AN, Engelman JA, Faber AC (2015). The BCL2 family: key mediators of the apoptotic response to targeted anticancer therapeutics. Cancer Discov.

[43] Bruns DR, Drake JC, Biela LM, Peelor FF, Miller BF, Hamilton KL (2015). Nrf2 signaling and the slowed aging phenotype: evidence from long-lived models. Oxid Med Cell Longev..

[44] Sirokmany G, Donko A, Geiszt M (2016). Nox/Duox family of NADPH oxidases: lessons from knockout mouse models. Trends Pharmacol Sci.

[45] Gunzel D, Yu AS (2013). Claudins and the modulation of tight junction permeability. Physiol Rev.

[46] Bahl V, Lin S, Xu N, Davis B, Wang YH, Talbot P (2012). Comparison of electronic cigarette refill fluid cytotoxicity using embryonic and adult models. Reprod Toxicol.

[47] Behar RZ, Davis B, Wang Y, Bahl V, Lin S, Talbot P (2014). Identification of toxicants in cinnamon-flavored electronic cigarette refill fluids. Toxicol In Vitro.

[48] Gerloff J, Sundar IK, Freter R, Sekera ER, Friedman AE, Robinson R (2017). Inflammatory response and barrier dysfunction by different e-cigarette flavoring chemicals identified by gas chromatography-mass spectrometry in e-liquids and e-vapors on human lung epithelial cells and fibroblasts. Appl In Vitro Toxicol.

[49] Behar RZ, Luo W, Lin SC, Wang Y, Valle J, Pankow JF (2016). Distribution, quantification and toxicity of cinnamaldehyde in electronic cigarette refill fluids and aerosols. Tob Control..

[50] Clapp PW, Pawlak EA, Lackey JT, Keating JE, Reeber SL, Glish GL (2017). Flavored e-cigarette liquids and cinnamaldehyde impair respiratory innate immune cell function. Am J Physiol Lung Cell Mol Physiol.

[51] Moses E, Wang T, Corbett S, Jackson GR, Drizik E, Perdomo C (2017). Molecular impact of electronic cigarette aerosol exposure in human bronchial epithelium. Toxicol Sci.

[52] The Universal Protein Resource (UniProt). https://www.uniprot.org/uniprot/Q8IVF4. Accessed 26 Mar 2020.

[53] Mukherjee I, Roy S, Chakrabarti S (2019). Identification of important effector proteins in the FOXJ1 transcriptional network associated with ciliogenesis and ciliary function. Front Genet.

[54] Ikegami K, Sato S, Nakamura K, Ostrowski LE, Setou M (2010). Tubulin polyglutamylation is essential for airway ciliary function through the regulation of beating asymmetry. Proc Natl Acad Sci USA.

[55] Tilley AE, Walters MS, Shaykhiev R, Crystal RG (2015). Cilia dysfunction in lung disease. Annu Rev Physiol.

[56] Farsalinos KE, Gillman G (2017). Carbonyl emissions in e-cigarette aerosol: a systematic review and methodological considerations. Front Physiol.

[57] Sun Y, Ito S, Nishio N, Tanaka Y, Chen N, Isobe K (2014). Acrolein induced both pulmonary inflammation and the death of lung epithelial cells. Toxicol Lett.

[58] Khlystov A, Samburova V (2016). Flavoring compounds dominate toxic aldehyde production during e-cigarette vaping. Environ Sci Technol.

[59] Cirillo S, Vivarelli F, Turrini E, Fimognari C, Burattini S, Falcieri E (2019). The customizable e-cigarette resistance influences toxicological outcomes: lung degeneration, inflammation and oxidative stress-induced in a rat model. Toxicol Sci.

[60] Stratton K, Kwan LY, Eaton DL. Committee on the review of the health effects of electronic nicotine delivery systems. Public health Consequences of e-cigarettes. A consensus study report of the National Academies of Sciences, Engineering, Medicine. 2018.

[61] Hubbs AF, Cumpston AM, Goldsmith WT, Battelli LA, Kashon ML, Jackson MC (2012). Respiratory and olfactory cytotoxicity of inhaled 2,3-pentanedione in Sprague-Dawley rats. Am J Pathol.

[62] Vas CA, Porter A, McAdam K (2019). Acetoin is a precursor to diacetyl in e-cigarette liquids. Food Chem Toxicol.

[63] Sassano MF, Davis ES, Keating JE, Zorn BT, Kochar TK, Wolfgang MC (2018). Evaluation of e-liquid toxicity using an open-source high-throughput screening assay. PLoS Biol..

[64] Muthumalage T, Prinz M, Ansah KO, Gerloff J, Sundar IK, Rahman I (2017). Inflammatory and oxidative responses induced by exposure to commonly used e-cigarette flavoring chemicals and flavored e-liquids without nicotine. Front Physiol.

[65] Holden LL, Truong L, Simonich MT, Tanguay RL (2020). Assessing the hazard of e-cigarette flavor mixtures using zebrafish. Food Chem Toxicol.

[66] Wavreil FDM, Heggland SJ (2020). Cinnamon-flavored electronic cigarette liquids and aerosols induce oxidative stress in human osteoblast-like MG-63 cells. Toxicol Rep.

[67] Lerner CA, Sundar IK, Yao H, Gerloff J, Ossip DJ, McIntosh S (2015). Vapors produced by electronic cigarettes and e-juices with flavorings induce toxicity, oxidative stress, and inflammatory response in lung epithelial cells and in mouse lung. PLoS ONE.

[68] Wigenstam E, Koch B, Bucht A, Jonasson S (2015). N-acetyl cysteine improves the effects of corticosteroids in a mouse model of chlorine-induced acute lung injury. Toxicology.

[69] Zhang M, Xia H, Yu M, Zhu L, Ju L, Chen J (2018). N-acetylcysteine prevents cytotoxic effects induced by man-made mineral fibers in a human bronchial epithelial cell line. Toxicol In Vitro.

[70] Sundar IK, Javed F, Romanos GE, Rahman I (2016). E-cigarettes and flavorings induce inflammatory and pro-senescence responses in oral epithelial cells and periodontal fibroblasts. Oncotarget.

[71] Ganapathy V, Manyanga J, Brame L, McGuire D, Sadhasivam B, Floyd E (2017). Electronic cigarette aerosols suppress cellular antioxidant defenses and induce significant oxidative DNA damage. PLoS ONE.

[72] Bitzer ZT, Goel R, Reilly SM, Elias RJ, Silakov A, Foulds J (2018). Effect of flavoring chemicals on free radical formation in electronic cigarette aerosols. Free Radic Biol Med.

[73] The Flavor and Extract Manugfacturers Association of the United States. Respiratory Health and Safety in the Flavor Manufacturing Workplace, 2012 update. April 2012 report.

[74] Allen JG, Flanigan SS, LeBlanc M, Vallarino J, MacNaughton P, Stewart JH (2016). Flavoring chemicals in e-cigarettes: diacetyl, 2,3-pentanedione, and acetoin in a sample of 51 products, including fruit-, candy, and cocktail-flavored e-cigarettes. Environ Health Perspect.

[75] Clapp PW, Lavrich KS, van Heusden CA, Lazarowski ER, Carson JL, Jaspers I (2019). Cinnamaldehyde in flavored e-cigarette liquids temporarily suppresses bronchial epithelial cell ciliary motility by dysregulation of mitochondrial function. Am J Physiol Lung Cell Mol Physiol.

[76] Lam HC, Cloonan SM, Bhashyam AR, Haspel JA, Singh A, Sathirapongsasuti JF (2013). Histone deacetylase 6–mediated selective autophagy regulates COPD-associated cilia dysfunction. J Clin Invest.

[77] Wolff RK (1986). Effects of airborne pollutants on mucociliary clearance. Environ Health Perspect.

[78] Chung S, Baumlin N, Dennis JS, Moore R, Salathe SF, Whitney PL (2019). Electronic cigarette vapor with nicotine causes airway mucociliary dysfunction preferentially via TRPA1 receptors. Am J Respir Crit Care Med.

[79] Braakhuis HM, He R, Vandebriel RJ, Gremmer ER, Zwart E, Vermeulen JP (2020). An air-liquid interface bronchial epithelial model for realistic, repeated inhalation exposure to airborne particles for toxicity testing. J Vis Exp..

[80] Herr C, Tsitouras K, Niederstaber J, Backes C, Beisswenger C, Dong L (2020). Cigarette smoke and electronic cigarettes differentially activate bronchial epithelial cells. Respir Res.

