# Supplementary material for: Sub-ohm vaping increases the levels of carbonyls, is cytotoxic, and alters gene expression in human bronchial epithelial cells exposed at the air–liquid interface
Source: Respir Res. 2020 Nov 19;21:305. doi: 10.1186/s12931-020-01571-1 (PMC7678293; doi:10.1186/s12931-020-01571-1)
Supplement: Supplementary file 1 — Additional file 1: Table S1. Primers used for qRT-PCR analysis of human genes. Figure S1. 3 days of butter-flavored e-cig aerosol exposure under sub-ohm conditions decrease viable cell numbers (trials #2 and 3). A) Numbers of viable cells were significantly decreased by the e-cig aerosol produced under sub-ohm vaping conditions (trial #2). B) Numbers of viable cells were decreased by the e-cig aerosol produced under sub-ohm vaping conditions (trial #3). Data are presented as mean ± SEM (n = 3 cell inserts per group). Comparisons between groups were made by the student t-test; *p < 0.05: significantly different from the other group. Figure S2. 1 day of butter-flavored e-cig aerosol exposure under sub-ohm conditions (0.15 Ω & 4.8 V) affects the integrity of H292 cells’ tight junctions (trial #2). H292 cells were exposed to butter-flavored e-cig aerosol at the air–liquid interface (ALI) for 1 day. A) Numbers of viable cells. B) Levels of extracellular lactate dehydrogenase (LDH). C) Levels of extracellular reactive oxygen species (ROS) were significantly increased by butter-flavored e-cig aerosol compared to the air control group. D) Levels of extracellular nitric oxide (NO). E) Exposure of cells to NAC pre-treatment and NAC pre-treatment plus butter-flavored e-cig aerosol had no significant effect on cellular viability. F) The transepithelial electrical resistance (TEER) values were significantly decreased by butter-flavored e-cig aerosols compared to the respective air control group. A-F: Data are presented as mean ± SEM (n = 3 cell inserts per group). For each cell insert, bioassays were further evaluated in duplicate or triplicate. For assays B – D, data were normalized to cell count. Comparisons between the e-cig group and the air control group were made by the student t-test; *p < 0.05: significantly different from air control; ξp < 0.05: significantly different from e-cig aerosol exposed-cells without NAC pre-treatment. Figure S3. 1 day of butter-flavored e-cig [file 12931_2020_1571_MOESM1_ESM.docx]

**Title:** Sub-ohm vaping increases the levels of carbonyls, is cytotoxic, and alters gene expression in human bronchial epithelial cells exposed at the air-liquid interface

### Authors: Alexandra Noël*^1^, Ekhtear Hossain^1^, Zakia Perveen^1^, Hasan Zaman^1^, and Arthur L. Penn^1^.

### Affiliations: ^1^Department of Comparative Biomedical Sciences, School of Veterinary Medicine, Louisiana State University, 1909 Skip Bertman Drive, Baton Rouge, LA, 70803, USA.

**Additional Information**

**Additional Table S1. Primers used for qRT-PCR analysis of human genes.**

| Gene | Gene primer sequences |
| --- | --- |
| α7nAChR | Forward – CGAAAGCGAGGCGGTCTGCAGCGAGTGGAA  Reverse – CGAAAGCGAGGCGGTCTGCAGCGAGTGGAA |
| ACTB | Forward – GGACCTGACTGACTACCTCAT  Reverse – CGTAGCACAGCTTCTCCTTAAT |
| AHR | Forward – TGGTCTCCCCCAGACAGTAG  Reverse – TTCATTGCCAGAAAACCAGA |
| ALDH-3A1 | Forward – TGTTCTCCAGCAACGACAAGG  Reverse – AGGGCAGAGAGTGCAAGGT |
| BCL-2 | Forward – TTGCCAGCCGGAACCTATG  Reverse – CGAAGGCGACCAGCAATGATA |
| CCL-2 | Forward – AGCATGAAAGTCTCTGCCGC  Reverse – GGCATTGATTGCATCTGGCTG |
| CLD-4 | Forward – TCCTGACTCACGGTGCAAAG  Reverse – CGTAGGATTCCAAGCGCTG |
| CYP-1A1 | Forward – TCGGCCACGGAGTTTCTTC  Reverse – GGTCAGCATGTGCCCAATCA |
| CYP-1B1 | Forward – AAGTTCTTGAGGCACTGCGAA  Reverse – GGCCGGTACGTTCTCCAAAT |
| IL-6 | Forward – ACTCACCTCTTCAGAACGAATTG  Reverse – CCATCTTTGGAAGGTTCAGGTTG |
| IL-8 | Forward – ACTGAGAGTGATTGAGAGTGGAC  Reverse – AACCCTCTGCACCCAGTTTTC |
| IL-13 | Forward – AAAGTTCAGGATATGGATTGCGT  Reverse – ACCTGGCATAGGTGTACTTCT |
| MMP-9 | Forward – TGTACCGCTATGGTTACACTCG  Reverse – GGCAGGGACAGTTGCTTCT |
| MMP-12 | Forward – GCATGGGCTAGGATTCCACC  Reverse – CATGAACCGTGAGGATGTTGA |
| NFKB-1 | Forward – CCTGAGACAAATGGGCTACAC  Reverse – TTTAGGGCTTTGGTTTACACGG |
| iNOS | Forward – TGGATGCAACCCCATTGTC  Reverse – CCCGCTGCCCCAGTTT |
| nNOS | Forward – CGGCATCACCAGGAAGAAGA  Reverse – CATGAGCGAGGCGGAGAT |
| SERPIN-A1A | Forward – TGAGGCACGATGGCAACAT  Reverse – GAGCCCTCTCTTGATCTGGG |
| SOD-1 | Forward – GGTGGGCCAAAGGATGAAGAG  Reverse – CCACAAGCCAAACGACTTCC |
| SOD-2 | Forward – GCTCCGGTTTTGGGGTATCTG  Reverse – GCGTTGATGTGAGGTTCCAG |
| SOD-3 | Forward – ATGCTGGCGCTACTGTGTTC  Reverse – CTCCGCCGAGTCAGAGTTG |
| STAT-3 | Forward – GGAGGAGTTGCAGCAAAAAG  Reverse - TGTGTTTGTGCCCAGAATGT |
| TNF-α | Forward – GCCACCACGCTCTTCTGT  Reverse – GGCTACGGGCTTGTCACTC |
| ZO-2 | Forward – TTGAAGACACGGACGGTGAA  Reverse – GTGATGGACGACACCAGCG |


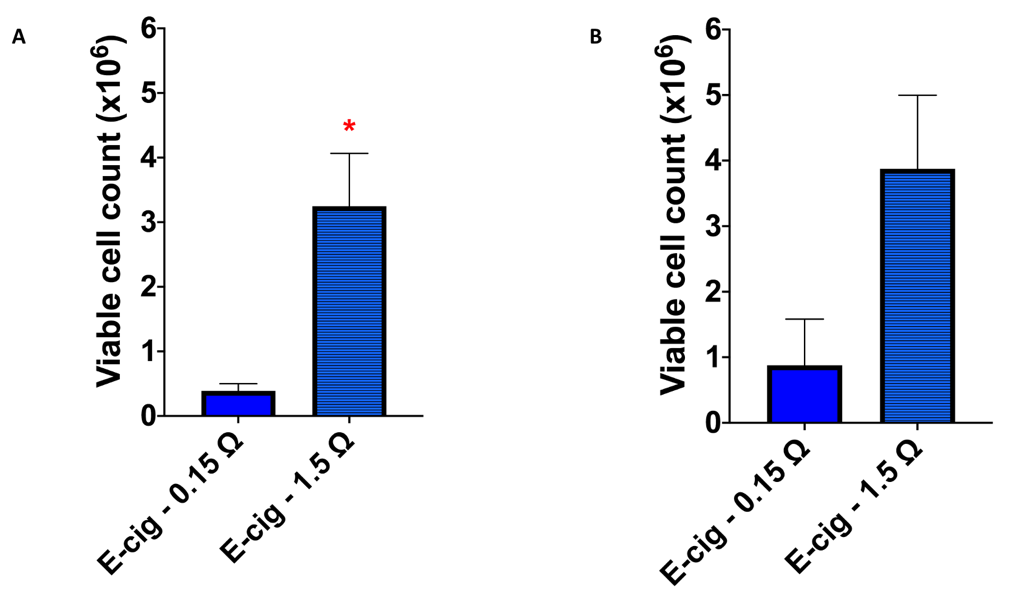


**Additional Figure S1**. **3 days of butter-flavored e-cig aerosol exposure under sub-ohm conditions decrease viable cell numbers (trials #2 and 3). A)** Numbers of viable cells were significantly decreased by the e-cig aerosol produced under sub-ohm vaping conditions (trial #2). **B)** Numbers of viable cells were decreased by the e-cig aerosol produced under sub-ohm vaping conditions (trial #3). Data are presented as mean ± SEM (n = 3 cell inserts per group). Comparisons between groups were made by the student t-test; **p* < 0.05: significantly different from the other group.


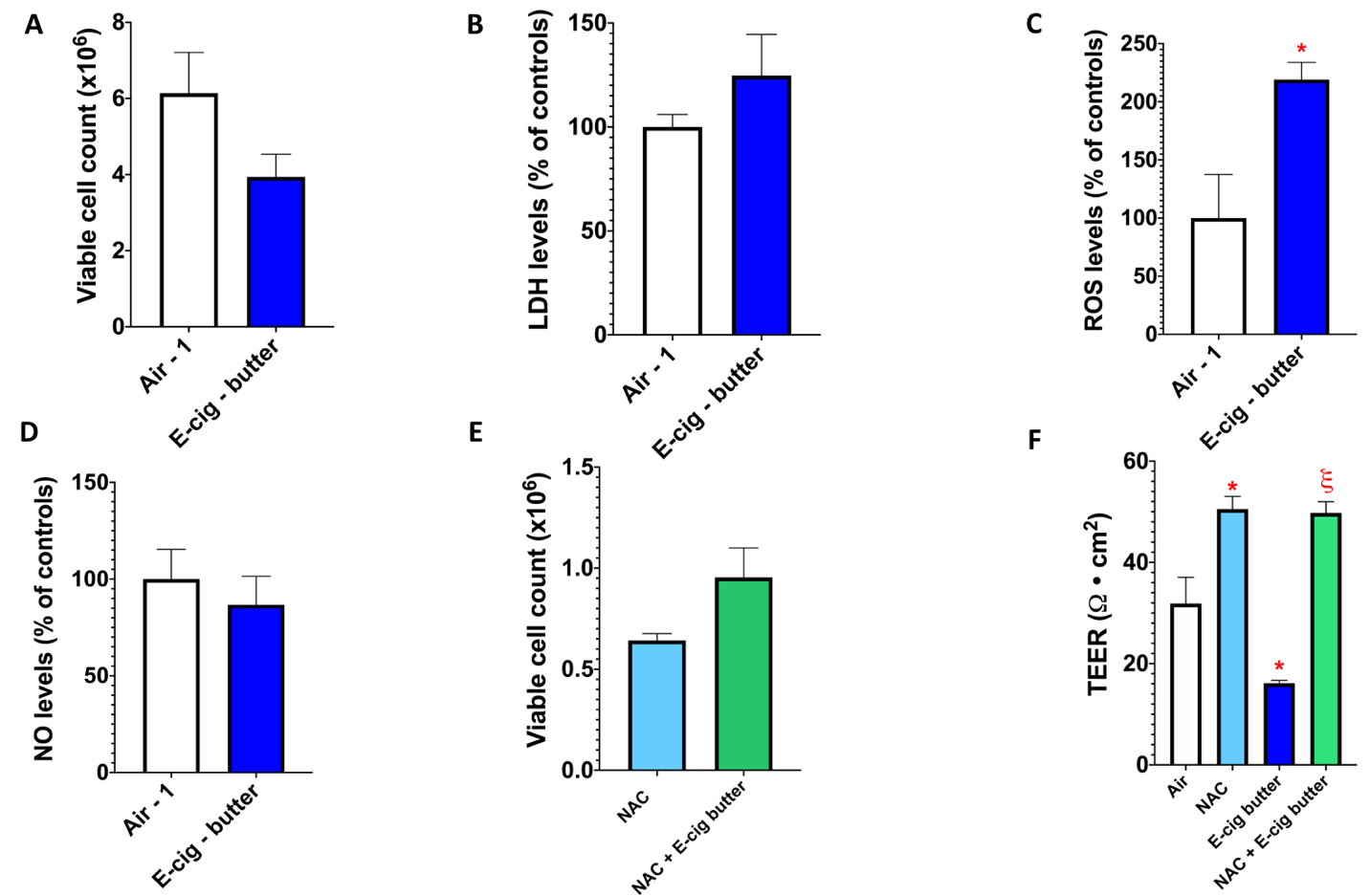


**Additional Figure S2. 1 day of butter-flavored e-cig aerosol exposure under sub-ohm conditions (0.15 Ω & 4.8 V) affects the integrity of H292 cells’ tight junctions (trial #2).** H292 cells were exposed to butter-flavored e-cig aerosol at the air-liquid interface (ALI) for 1 day. **A)** Numbers of viable cells. **B)** Levels of extracellular lactate dehydrogenase (LDH). **C)** Levels of extracellular reactive oxygen species (ROS) were significantly increased by butter-flavored e-cig aerosol compared to the air control group. **D)** Levels of extracellular nitric oxide (NO). **E)** Exposure of cells to NAC pre-treatment and NAC pre-treatment plus butter-flavored e-cig aerosol had no significant effect on cellular viability. **F)** The transepithelial electrical resistance (TEER) values were significantly decreased by butter-flavored e-cig aerosols compared to the respective air control group. **A-F:** Data are presented as mean ± SEM (n = 3 cell inserts per group). For each cell insert, bioassays were further evaluated in duplicate or triplicate. For assays **B** – **D**, data were normalized to cell count. Comparisons between the e-cig group and the air control group were made by the student t-test; **p* < 0.05: significantly different from air control; ^ξ^*p* < 0.05: significantly different from e-cig aerosol exposed-cells without NAC pre-treatment.


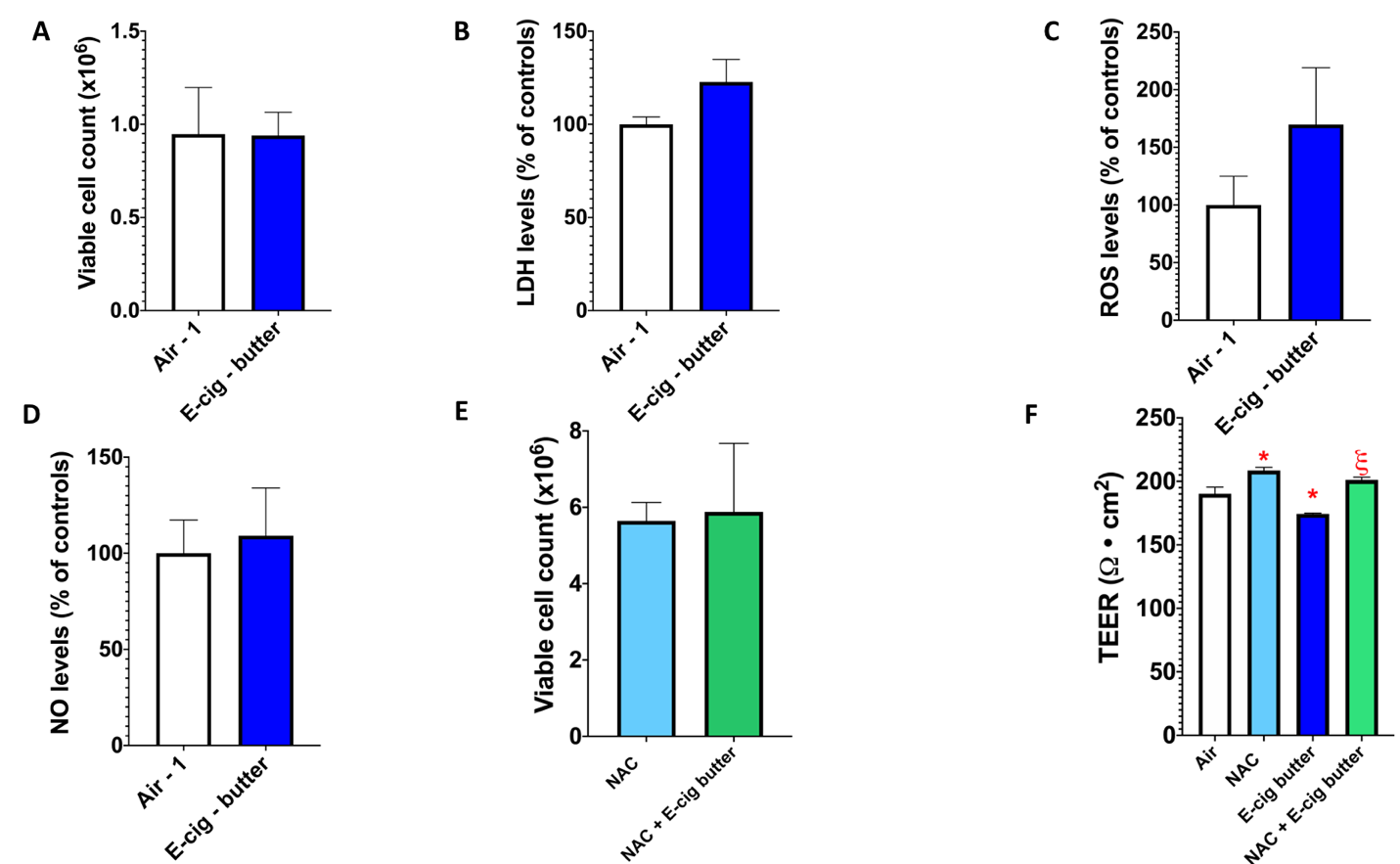


**Additional Figure S3. 1 day of butter-flavored e-cig aerosol exposure under sub-ohm conditions (0.15 Ω & 4.8 V) affects the integrity of H292 cells’ tight junctions (trial #3).** H292 cells were exposed to butter-flavored e-cig aerosol at the air-liquid interface (ALI) for 1 day. **A)** Numbers of viable cells. **B)** Levels of extracellular lactate dehydrogenase (LDH). **C)** Levels of extracellular reactive oxygen species (ROS). **D)** Levels of extracellular nitric oxide (NO). **E)** Exposure of cells to NAC pre-treatment and NAC pre-treatment plus butter-flavored e-cig aerosol had no significant effect on cellular viability. **F)** The transepithelial electrical resistance (TEER) values were significantly decreased by butter-flavored e-cig aerosols compared to the respective air control group. **A-F:** Data are presented as mean ± SEM (n = 3 cell inserts per group). For each cell insert, bioassays were further evaluated in duplicate or triplicate. For assays **B** – **D**, data were normalized to cell count. Comparisons between the e-cig group and the air control group were made by the student t-test; **p* < 0.05: significantly different from air control; ^ξ^*p* < 0.05: significantly different from e-cig aerosol exposed-cells without NAC pre-treatment.

**
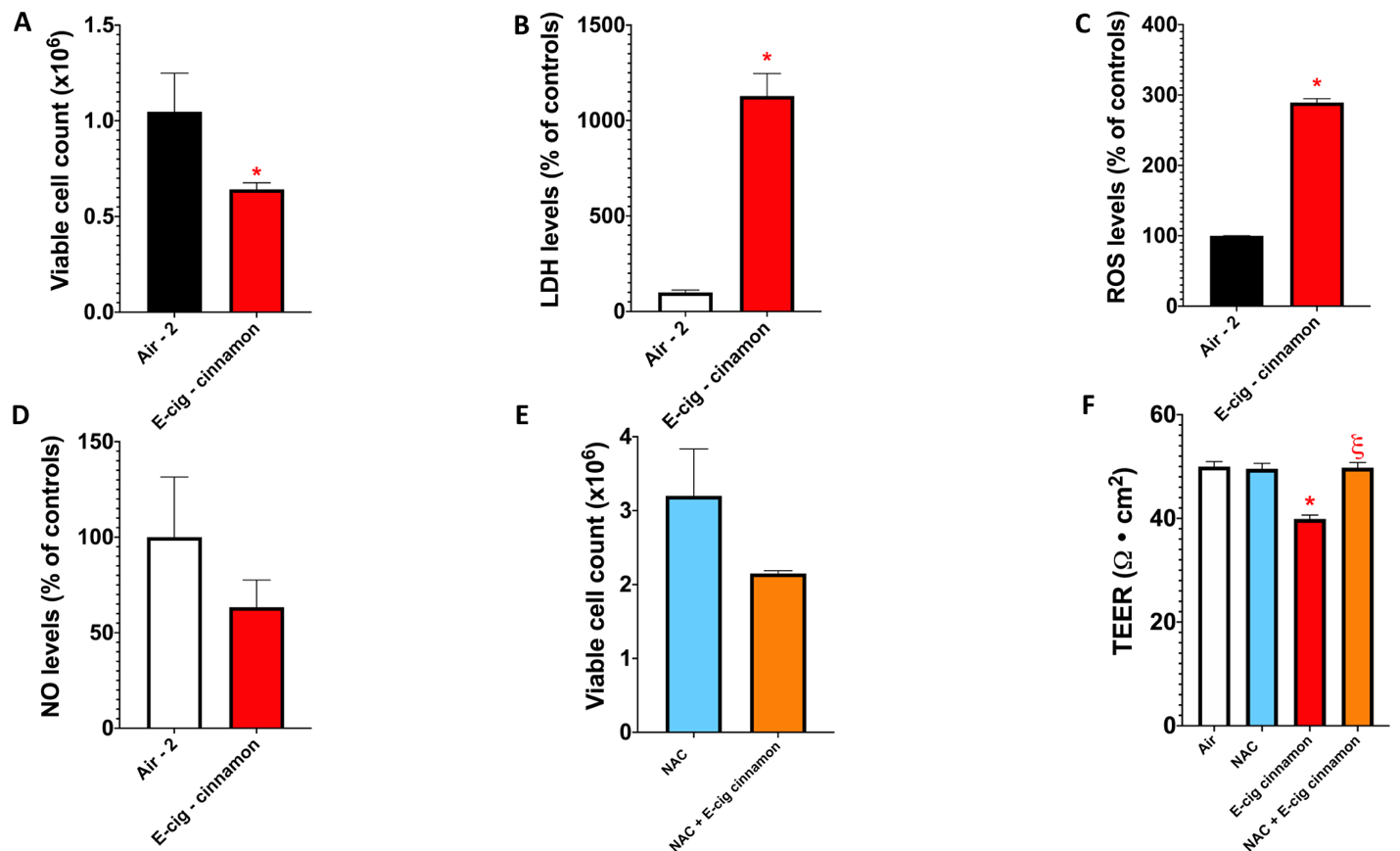
**

**Additional Figure S4. 1 day of cinnamon-flavored e-cig aerosol exposure under sub-ohm conditions (0.15 Ω & 4.8 V) decreases cell viability and affects the integrity of H292 cells’ tight junctions (trial #2).** H292 cells were exposed to cinnamon-flavored e-cig aerosol at the air-liquid interface (ALI) for 1 day. **A)** Numbers of viable cells were significantly decreased by the cinnamon-flavored e-cig aerosol compared to air control. **B)** Levels of extracellular lactate dehydrogenase (LDH) were significantly increased by cinnamon-flavored e-cig aerosols compared to the air control group. **C)** Levels of extracellular reactive oxygen species (ROS) were significantly increased by cinnamon-flavored e-cig aerosols compared to the air control group. **D)** Levels of extracellular nitric oxide (NO). **E)** Exposure of cells to NAC pre-treatment and NAC pre-treatment plus cinnamon-flavored e-cig aerosol had no significant effect on cellular viability. **F)** The transepithelial electrical resistance (TEER) values were significantly decreased by cinnamon-flavored e-cig aerosols compared to the air control group. **A-F:** Data are presented as mean ± SEM (n = 3 cell inserts per group). For each cell insert, bioassays were further evaluated in duplicate or triplicate. For assays **B** - **D** data were normalized to cell count. Comparisons between the e-cig group and the air control group were made by the student t-test; **p* < 0.05: significantly different from air control; ^ξ^*p* < 0.05: significantly different from e-cig aerosol exposed-cells without NAC pre-treatment.


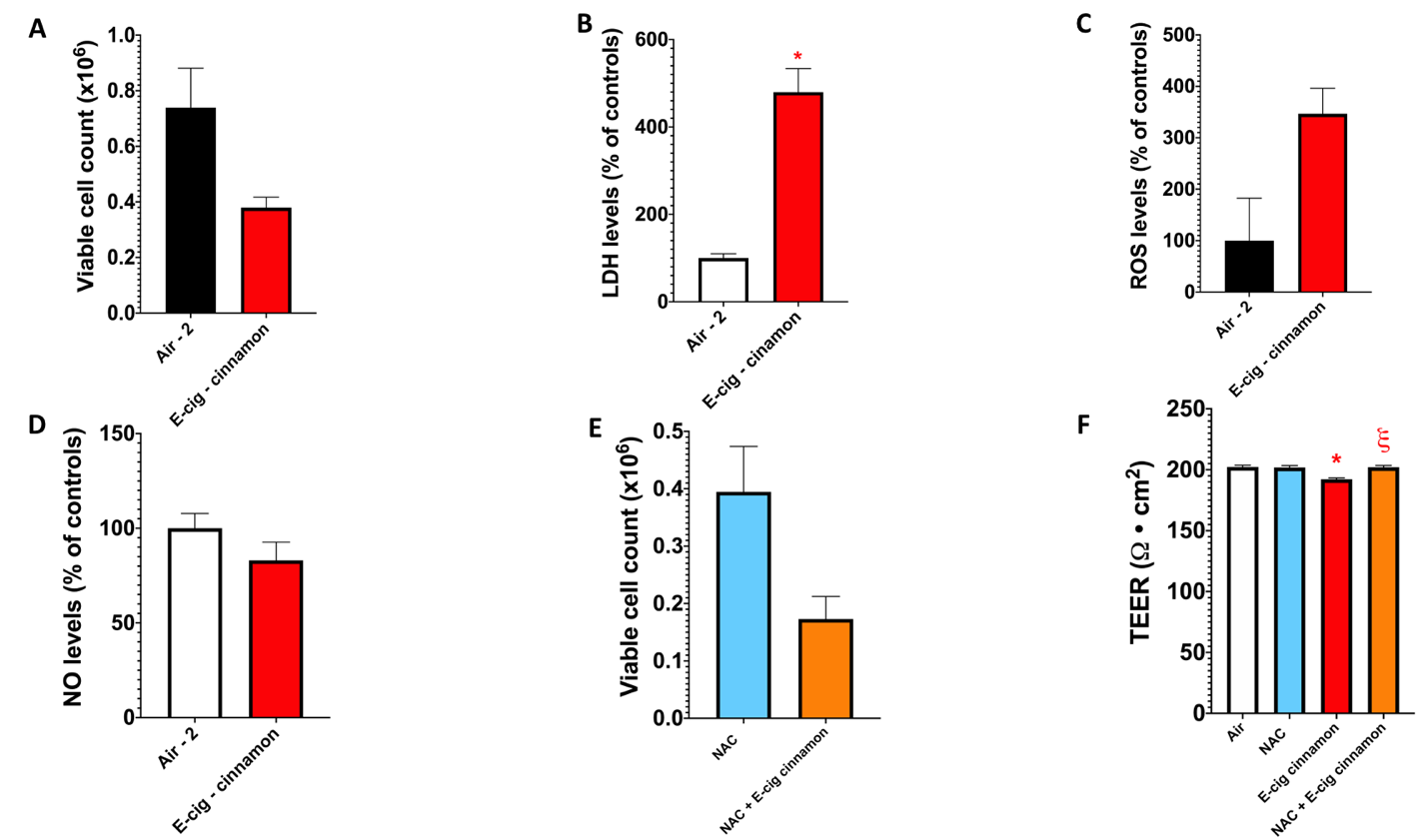


**Additional Figure S5. 1 day of cinnamon-flavored e-cig aerosol exposure under sub-ohm conditions (0.15 Ω & 4.8 V) decreases cell viability and affects the integrity of H292 cells’ tight junctions (trial #3).** H292 cells were exposed to cinnamon-flavored e-cig aerosol at the air-liquid interface (ALI) for 1 day. **A)** Numbers of viable cells. **B)** Levels of extracellular lactate dehydrogenase (LDH) were significantly increased by cinnamon-flavored e-cig aerosols compared to the air control group. **C)** Levels of extracellular reactive oxygen species (ROS). **D)** Levels of extracellular nitric oxide (NO). **E)** Exposure of cells to NAC pre-treatment and NAC pre-treatment plus cinnamon-flavored e-cig aerosol had no significant effect on cellular viability. **F)** The transepithelial electrical resistance (TEER) values were significantly decreased by cinnamon-flavored e-cig aerosols compared to the air control group. **A-F:** Data are presented as mean ± SEM (n = 3 cell inserts per group). For each cell insert, bioassays were further evaluated in duplicate or triplicate. For assays **B –** **D**, data were normalized to cell count. Comparisons between the e-cig group and the air control group were made by the student t-test; **p* < 0.05: significantly different from air control.

**
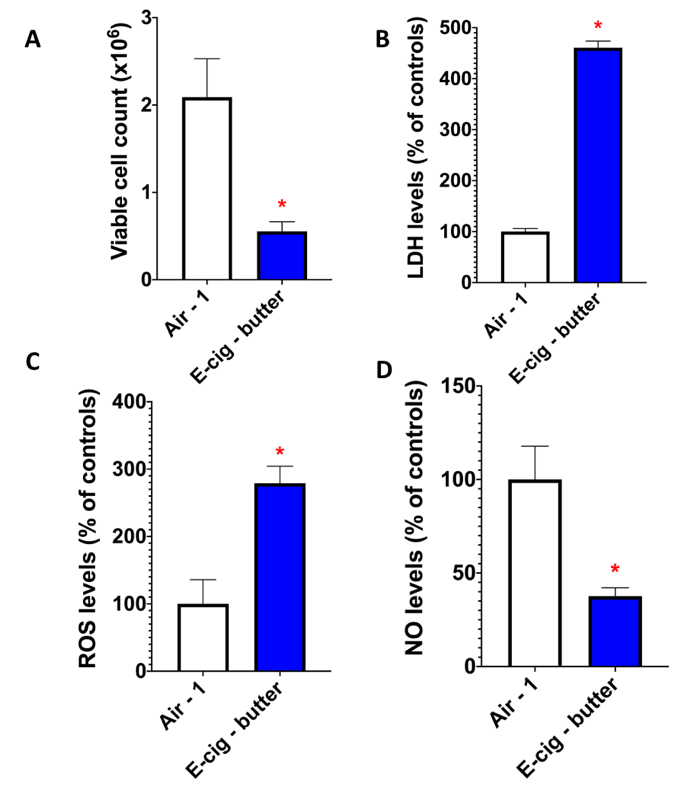
**

**Additional Figure S6. 3 days of butter-flavored e-cig aerosol exposure under sub-ohm conditions (0.15 Ω & 4.8 V) is cytotoxic and causes oxidative damage to H292 cells (trial #2).** H292 cells were exposed to butter-flavored e-cig aerosol at the air-liquid interface (ALI) for 3 days. **A)** Numbers of viable cells were significantly decreased by butter-flavored e-cig aerosol compared to air controls. **B)** Levels of extracellular lactate dehydrogenase (LDH) were significantly increased by butter-flavored e-cig aerosol compared to air controls. **C)** Levels of extracellular reactive oxygen species (ROS) were significantly increased by butter-flavored e-cig aerosol compared to air controls. **D)** Levels of extracellular nitric oxide (NO) were significantly decreased by butter-flavored e-cig aerosol compared to air controls. **A-D:** Data are presented as mean ± SEM (n = 3 cell inserts per group). For each cell insert, bioassays were further evaluated in duplicate or triplicate. For assays **B – D**, data were normalized to cell count. Comparisons between the e-cig group and the air control group were made by the student t-test; **p* < 0.05: significantly different from air control.

**
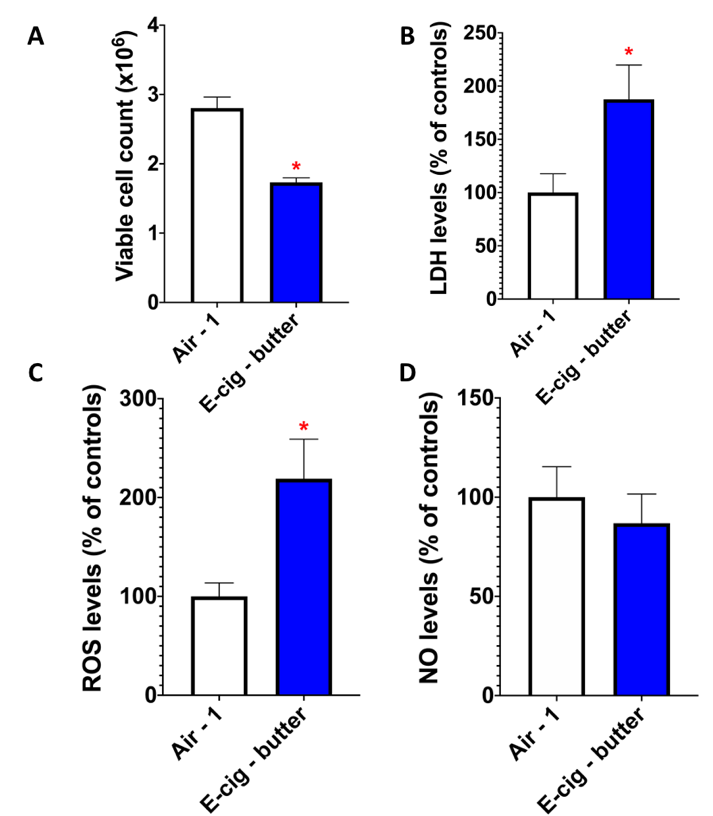
**

**Additional Figure S7. 3 days of butter-flavored e-cig aerosol exposure under sub-ohm conditions (0.15 Ω & 4.8 V) is cytotoxic and causes oxidative damage to H292 cells (trial #3).** H292 cells were exposed to butter-flavored e-cig aerosol at the air-liquid interface (ALI) for 3 days. **A)** Numbers of viable cells were significantly decreased by butter-flavored e-cig aerosol compared to air controls. **B)** Levels of extracellular lactate dehydrogenase (LDH) were significantly increased by butter-flavored e-cig aerosol compared to air controls. **C)** Levels of extracellular reactive oxygen species (ROS) were significantly increased by butter-flavored e-cig aerosol compared to air controls. **D)** Levels of extracellular nitric oxide (NO). **A-D:** Data are presented as mean ± SEM (n = 3 cell inserts per group). For each cell insert, bioassays were further evaluated in duplicate or triplicate. For assays **B - D**, data were normalized to cell count. Comparisons between the e-cig group and the air control group were made by the student t-test; **p* < 0.05: significantly different from air control.

**
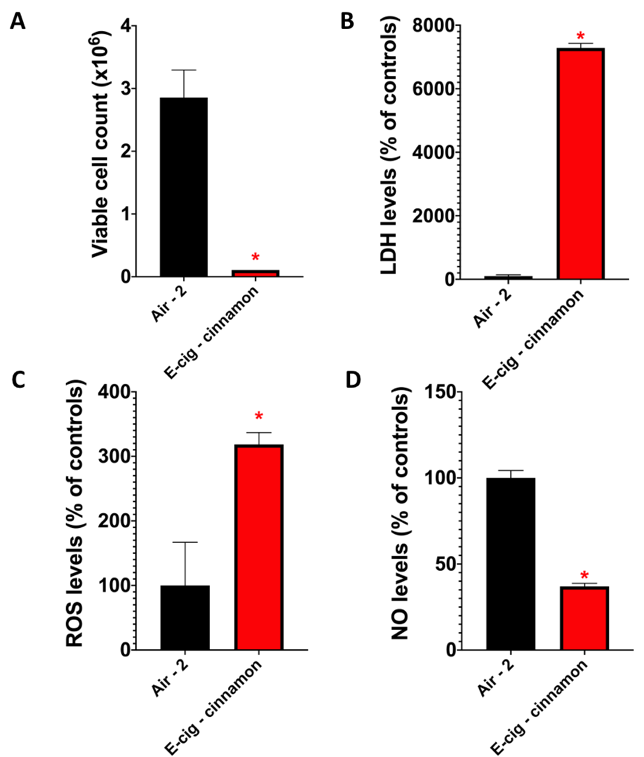
**

**Additional Figure S8. 3 days of cinnamon-flavored e-cig aerosol exposure under sub-ohm conditions (0.15 Ω & 4.8 V) is cytotoxic and causes oxidative damage to H292 cells (trial #2).** H292 cells were exposed to cinnamon-flavored e-cig aerosol at the air-liquid interface (ALI) for 3 days. **A)** Numbers of viable cells were significantly decreased by cinnamon-flavored e-cig aerosol compared to air controls. **B)** Levels of extracellular lactate dehydrogenase (LDH) were significantly increased by cinnamon-flavored e-cig aerosol compared to air controls. **C)** Levels of extracellular reactive oxygen species (ROS) were significantly increased by cinnamon-flavored e-cig aerosol compared to air controls. **D)** Levels of extracellular nitric oxide (NO) were significantly decreased by cinnamon-flavored e-cig aerosol compared to air controls. **A-D:** Data are presented as mean ± SEM (n = 3 cell inserts per group). For each cell insert, bioassays were further evaluated in duplicate or triplicate. For assays **B - D**, data were normalized to cell count. Comparisons between the e-cig group and the air control group were made by the student t-test; **p* < 0.05: significantly different from air control.

**
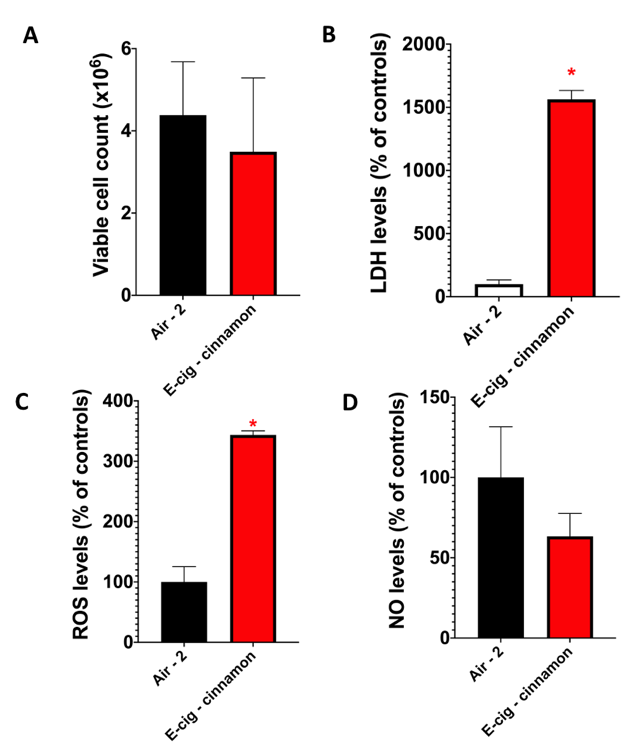
**

**Additional Figure S9. 3 days of cinnamon-flavored e-cig aerosol exposure under sub-ohm conditions (0.15 Ω & 4.8 V) is cytotoxic and causes oxidative damage to H292 cells (trial #3).** H292 cells were exposed to cinnamon-flavored e-cig aerosol at the air-liquid interface (ALI) for 3 days. **A)** Numbers of viable cells. **B)** Levels of extracellular lactate dehydrogenase (LDH) were significantly increased by cinnamon-flavored e-cig aerosol compared to air controls. **C)** Levels of extracellular reactive oxygen species (ROS) were significantly increased by cinnamon-flavored e-cig aerosol compared to air controls. **D)** Levels of extracellular nitric oxide (NO). **A-D:** Data are presented as mean ± SEM (n = 3 cell inserts per group). For each cell insert, bioassays were further evaluated in duplicate or triplicate. For assays **B - D**, data were normalized to cell count. Comparisons between the e-cig group and the air control group were made by the student t-test; **p* < 0.05: significantly different from air control.
